# Cold stress induces enhanced chromatin accessibility and bivalent histone modifications H3K4me3 and H3K27me3 of active genes in potato

**DOI:** 10.1186/s13059-019-1731-2

**Published:** 2019-06-17

**Authors:** Zixian Zeng, Wenli Zhang, Alexandre P. Marand, Bo Zhu, C. Robin Buell, Jiming Jiang

**Affiliations:** 1 Department of Horticulture, University of Wisconsin-Madison, Madison, WI 53706 USA; 2 Department of Plant Biology, Michigan State University, East Lansing, MI 48824 USA; 3 Department of Horticulture, Michigan State University, East Lansing, MI 48824 USA; 4 Department of Biological Science, College of Life Sciences, Sichuan Normal University, Chengdu, 610101 Sichuan China; 5 State Key Laboratory for Crop Genetics and Germplasm Enhancement, Nanjing Agriculture University, Nanjing, 210095 Jiangsu China; 6 Michigan State University AgBioResearch, East Lansing, MI 48824 USA

**Keywords:** Potato, Cold stress, DNase I hypersensitive site, Open chromatin, Bivalent mark, H3K4me3, H3K27me3

## Abstract

**Background:**

Cold stress can greatly affect plant growth and development. Plants have developed special systems to respond to and tolerate cold stress. While plant scientists have discovered numerous genes involved in responses to cold stress, few studies have been dedicated to investigation of genome-wide chromatin dynamics induced by cold or other abiotic stresses.

**Results:**

Genomic regions containing active *cis*-regulatory DNA elements can be identified as DNase I hypersensitive sites (DHSs). We develop high-resolution DHS maps in potato (*Solanum tuberosum*) using chromatin isolated from tubers stored under room (22 °C) and cold (4 °C) conditions. We find that cold stress induces a large number of DHSs enriched in genic regions which are frequently associated with differential gene expression in response to temperature variation. Surprisingly, active genes show enhanced chromatin accessibility upon cold stress. A large number of active genes in cold-stored tubers are associated with the bivalent H3K4me3-H3K27me3 mark in gene body regions. Interestingly, upregulated genes associated with the bivalent mark are involved in stress response, whereas downregulated genes with the bivalent mark are involved in developmental processes. In addition, we observe that the bivalent mark-associated genes are more accessible than others upon cold stress.

**Conclusions:**

Collectively, our results suggest that cold stress induces enhanced chromatin accessibility and bivalent histone modifications of active genes. We hypothesize that in cold-stored tubers, the bivalent H3K4me3-H3K27me3 mark represents a distinct chromatin environment with greater accessibility, which may facilitate the access of regulatory proteins required for gene upregulation or downregulation in response to cold stress.

**Electronic supplementary material:**

The online version of this article (10.1186/s13059-019-1731-2) contains supplementary material, which is available to authorized users.

## Background

Genomic regions, when bound by regulatory proteins, are thought to be depleted of nucleosomes or have undergone dynamic nucleosome modifications or displacement [1, 2]. These regions, also known as “open chromatin,” are highly enriched with active *cis-*regulatory DNA elements (CREs) in eukaryotic genomes [3, 4]. Open chromatin shows a pronounced sensitivity to cleavage by various nucleases, including deoxyribonuclease I (DNase I) [5, 6] and transposase Tn5 [7–9], whereas chromatin with DNA tightly bound by nucleosomes is drastically less sensitive to these nucleases. These genomic regions are known as DNase I hypersensitive sites (DHSs) and can be identified by DNase-seq [3, 10, 11] or ATAC-seq [8, 9]. DHSs have been shown to be frequently associated with most common types of active CREs, including promoters and enhancers [12, 13]. Strikingly, reporter assays indicated that 70–80% of the DHSs located in intergenic regions of *Arabidopsis thaliana* and *Zea mays* genomes show enhancer function [14, 15].

Histone modification plays an important role in epigenetic regulation of genes in response to environmental and developmental cues [16–18]. In plants, H3K4me3 is a euchromatin mark mainly distributed at 5′ ends of actively expressed genes [19–21]. These genes are generally associated with low levels of tissue specificity [19]. In contrast, H3K27me3 is associated with one of the major gene silencing systems in plants and is enriched across the transcribed regions of genes that are involved in many developmental and other processes [22]. Genes marked by H3K27me3 are usually transcriptionally inactive and display tissue specificity [22]. Genome-wide distributions of these histone modifications and their association with gene expression have been well-documented in a number of plant species [23, 24]. However, investigation of histone modification dynamics under abiotic stresses has been focused on individual stress-responsive genes. For instance, H3K4me3 has been found to be enriched at 5′ ends of the dehydration-induced genes in both *Arabidopsis* [25] and rice [26] as a response to dehydration. The enrichment of H3K4me3 in these genes was correlated with an increase in expression level [25, 26]. Nucleation and spreading of H3K27me3 in the transcribed region of the *FLOWERING LOCUS C* (*FLC*) can be induced by prolonged cold exposure [27]. The cold-induced deposition of H3K27me3 represses the expression of *FLC* for timely flowering [27].

Chromatin associated with both active and repressive histone modifications on the same nucleosomes were described as bivalent domains [28]. Bivalent domains marked by H3K4me3 and H3K27me3 were first discovered in mouse embryonic stem cells (ESCs) [28, 29]. In the mammalian systems, H3K4me3-H3K27me3 bivalent domains mainly overlap with the promoters of lineage-specific genes in ESCs, which are expressed at low levels to maintain pluripotency of ESCs [28, 29]. The H3K4me3 mark in the bivalent domains was proposed to poise genes for activation while H3K27me3 keeps them repressed [28, 30]. In plants, the presence and function of bivalent H3K4me3-H3K27me3 mark remain to be elucidated. There have been only sporadic reports on bivalent histone modifications in plants. For example, Berr et al. [31] reported that two genes involved in gametophyte development in *Arabidopsis* were associated with bivalent H3K4me3-H3K27me3 mark. The existence of bivalent H3K4me3-H3K27me3 mark was detected in a few genomic regions in *Arabidopsis* seedlings [32]. Under dehydration stress, both H3K4me3 and H3K27me3 were found to be enriched in the same regions of a few stress memory genes [33], suggesting the stress may trigger the deposition of the bivalent mark. These studies, however, were limited to a few genes or genomic regions and did not exclude potential artifacts, such as the deposition of H3K4me3 and H3K27me3 in different subpopulations of cells.

Potato (*Solanum tuberosum*) is the most important non-grain food crop and serves more than a billion people worldwide (http://cipotato.org/potato). Potato tubers must be stored at cold temperatures to prevent sprouting and to minimize disease losses. Unfortunately, cold storage triggers accumulation of reducing sugars, which is called cold-induced sweetening (CIS) [34]. CIS is the most pressing and long-lasting challenge to the potato industry; production of reducing sugars results in an unsatisfactory dark color and accumulation of acrylamide—a potential carcinogen—in processing potato products, including French fries and potato chips [35–37]. We are interested in chromatin dynamics associated with cold-stored tubers with a long-term goal to understand potato gene expression and regulation during cold storage. We developed high-resolution DHS maps in potato using chromatin isolated from tubers stored at room and cold conditions. We discovered that active genes (transcribed in both conditions) surprisingly displayed enhanced chromatin accessibility upon cold storage. A large number of active genes were associated with bivalent histone modifications of H3K4me3 and H3K27me3 upon cold storage. Upregulated genes associated with the bivalent mark were enriched in functions related to stress response, while the downregulated genes associated with the bivalent mark were involved in developmental processes. We hypothesize that the bivalent H3K4me3-H3K27me3 mark represents a distinct chromatin environment with greater accessibility, which may facilitate the access of regulatory proteins required for gene upregulation or downregulation in response to the cold stress in potato tubers.

## Results

### Genome-wide identification of DHSs and their distribution in the potato genome

We constructed a total of six DNase-seq libraries (see the “Methods” section), composed of two biological replicates derived from tubers stored under room temperature (designated as RT tubers), tubers stored under 4 °C (designated as cold tubers), and leaf tissue (designated as leaves), respectively. Approximately, 45% (79/174 million [M]), 46% (84/183 M), and 51% (84/164 M) of the DNase-seq reads were mapped to unique positions in the potato genome sequence (Genome assembly DM v4.04 [38]) for RT tubers, cold tubers, and leaves, respectively (Additional file 2: Table S1). The coverage distribution between biological replicates for each tissue was highly correlated (Additional file 1: Figure S1). DHSs were identified using an in-house developed software, Popera (see the “Methods” section), with a false discovery rate (FDR) at 5%. Only consensus DHSs, identified in both biological replicates independently, were retained for downstream analyses. A total of 50,531 DHSs from RT tubers, 61,986 from cold tubers, and 81,603 from leaves were identified across the potato genome. DHSs were enriched toward the distal regions of each chromosome but depleted in centromeric regions (Additional file 1: Figure S2). The density of potato DHSs was positively correlated with gene density (Pearson’s correlation *r* = 0.82, *p* < 2.2e−16) and negatively correlated with transposable elements (Pearson’s correlation *r* = − 0.68, *p* < 2.2e−16) across all potato chromosomes (Additional file 1: Figure S3).

**Fig. 1:**
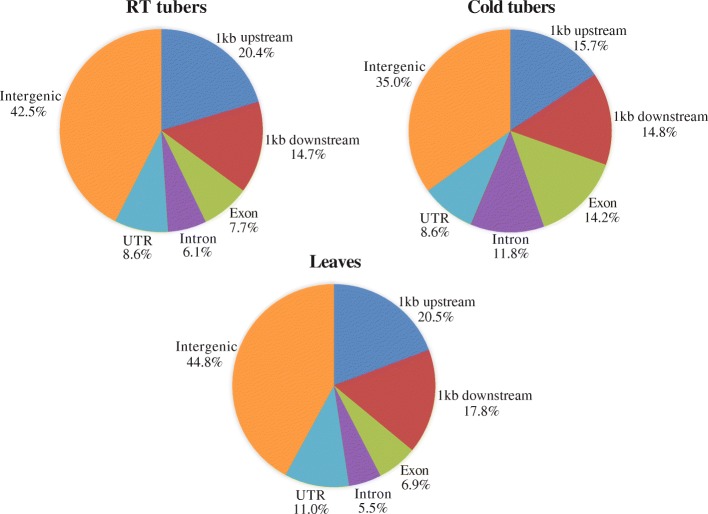
Distribution of DHSs in the potato genome. Consensus DHSs identified from two biological replicates of RT tubers (50,531), cold tubers (61,986), and leaves (81,603) were scored based on their positions relative to annotated potato genes (DM v4.04). The position of DHSs relative to genomic features was determined if the most frequent cutting site (a single base pair position) within a DHS overlaps with a genomic feature. 1 kb upstream indicates 1 kb regions upstream of TSSs. 1 kb downstream indicates 1 kb regions downstream of TTSs. Intergenic refers to regions greater than 1 kb away from any TSS or TTS

We then analyzed the locations of DHSs relative to annotated genes in the potato genome (see the “Methods” section). Approximately 20.4% of DHSs from RT tubers were mapped within 1 kb upstream of transcription start sites (TSSs) (Fig. 1). A large proportion (42.5%) of DHSs were located in intergenic regions (defined as at least 1 kb away from any TSS or transcription termination site (TTS)). A similar DHS distribution pattern was observed for leaves (Fig. 1). Interestingly, we found marked differences in the distribution of DHSs between cold and RT tubers. The proportion of DHSs identified in exons (14.2%) and introns (11.8%) from cold tubers was nearly double the proportion of DHSs in RT tubers (7.7% in exons and 6.1% in introns) (*z* test, *p* < 0.0002 for both exons and introns) (Fig. 1). There were also a lower proportion of intergenic DHSs in cold tubers (35.0%) versus RT tubers (42.5%) (*z* test, *p* < 0.0002).

### DHSs in genic regions and their association with differential gene expression

We found that more than half (31,089/61,986) of DHSs from cold tubers were unique to the cold condition. Strikingly, 38.5% (11,983) of the cold tuber-specific DHSs were located in exons (20.0%, 6228 DHSs) and introns (18.5%, 5755 DHSs) of 7314 genes. In contrast, 42.4% (21,430/50,531) of DHSs from RT tubers were RT tuber-specific, but only 14.5% (3114) of the RT tuber-specific DHSs were located in exons (7.0%, 1500) and introns (7.5%, 1614) of 2857 genes (Additional file 1: Figure S4). Thus, cold stress was associated with a nearly fourfold increase in the number of genic DHSs relative to RT condition.

**Fig. 2:**
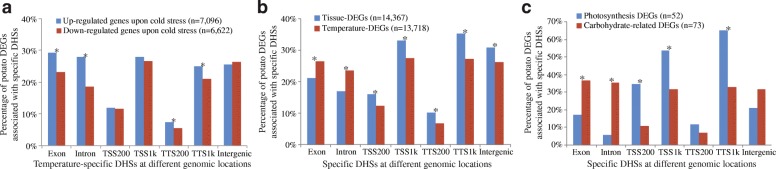
Comparison of differentially expressed genes (DEGs) associated with tissue- and temperature-specific DHSs. **a** The percentage of DEGs between RT and cold tubers associated with temperature-specific DHSs at different genomic locations, including exons, introns, TSS1k (1 kb upstream regions of TSSs), TSS200 (200 bp upstream regions of TSSs), TTS1k (1 kb downstream regions of TTSs), TTS200 (200 bp downstream regions of TTSs), and intergenic regions (1–5 kb upstream regions of TSSs or 1–5 kb downstream regions TTSs). **b** Comparison between potato tissue-DEGs (leaves vs. RT tubers) associated with tissue-specific DHSs and temperature-DEGs (RT tubers vs. cold tubers) associated with temperature-specific DHSs at different genomic locations. **c** Comparison of potato photosynthetic DEGs associated with tissue-specific DHSs and carbohydrate pathway-related DEGs associated with temperature-specific DHSs at different genomic locations. *z test *p* < 0.0002

We then investigated the potential association between temperature-specific DHSs (both RT- and cold tuber-specific DHSs) and differential gene expression detected using RNA-seq data from RT and cold tubers (Additional file 2: Table S2, Additional file 1: Figure S5). Among the 7314 genes harboring cold tuber-specific DHSs in exons and introns, over 69% were differentially expressed and generally upregulated in cold tubers (median fold change of 1.7, Wilcoxon rank sum test, *p* = 1.15e−15). Similarly, 68% of the genes (2857) harboring RT tuber-specific DHSs in exons and introns were differentially expressed and associated with a higher level of expression in RT tubers (median fold change of 1.5, Wilcoxon rank sum test, *p* = 2e−4). We found a total of 7096 upregulated and 6622 downregulated genes in cold-treated tubers comparing to RT tubers. Upregulated genes were more frequently associated with temperature-specific DHSs located in exons and introns compared to downregulated genes (*z* test, *p* < 0.0002) (Fig. 2a). By contrast, up- and downregulated genes showed a similar frequency of association with temperature-specific DHSs located in putative promoters (1 kb upstream of the TSS) and intergenic regions (Fig. 2a). These results suggest that temperature-induced DHS changes underlying genic regions are frequently associated with variation in gene expression.

We collected all differentially expressed genes (DEGs) between RT tubers and leaves (referred to tissue-DEGs hereafter) (Additional file 2: Table S2) and temperature-DEGs (between RT tubers and cold tubers). We then grouped these DEGs by the locations of their cognate DHSs. This allowed us to assess the relative impact of the genomic context underlying DHSs on differential gene expression associated with either tissue specificity or temperature response. DHSs located in genic regions were enriched for DEGs associated with temperature-specific expression (*z* test, *p* < 0.0002) (Fig. 2b). By contrast, DHSs residing in non-genic regions were more significantly associated with tissue-specific expression (*z* test, *p* < 0.0002) (Fig. 2b). Collectively, these results suggest that DHSs located in exons and introns are more likely to be associated with differential gene expression in response to temperature variation.

To further validate the genome-wide pattern, we selected a set of 73 genes related to carbohydrate metabolism [39] that were differentially expressed between RT and cold tubers (Additional file 2: Table S3). Genes in the carbohydrate metabolism pathway and their response to cold stress have been well-documented in potato [34]. Thus, these carbohydrate-related genes can represent those that respond to cold stress. Similarly, we selected 52 DEGs from the photosynthetic pathway to represent tissue specificity (Additional file 2: Table S4), reasoning that photosynthetic genes are only active in leaves since the potato tuber is not a primarily photosynthetic organ [40]. We then compared the DHS patterns found in photosynthetic genes to those found in the carbohydrate-related genes. We found that 17% (9/52) and 6% (3/52) of photosynthetic genes contained tissue-specific DHSs in exons and introns, respectively. By contrast, 37% (27/73) and 36% (26/73) of the carbohydrate-related genes contained temperature-specific DHSs in exons and introns, respectively, which are significantly more (*z* test, *p* < 0.0002) than photosynthetic genes (Fig. 2c). Moreover, tissue-specific DHSs located 1 kb up- and downstream of TSSs and TTSs were associated with a higher proportion of photosynthetic genes compared to carbohydrate-related genes (*z* test, *p* < 0.0002) (Fig. 2c). These results are consistent with the genome-wide pattern of the association between the genic DHSs and the temperature-DEGs in response to cold stress.

### Motifs associated with cold-specific DHSs

**Fig. 3:**
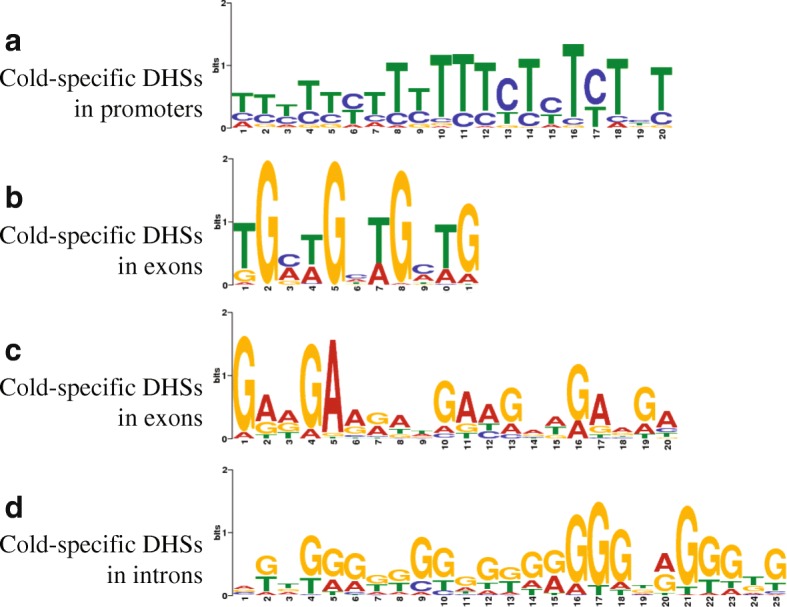
Enriched sequence motifs identified from cold-specific DHSs. **a** The top enriched motif in the cold-specific DHSs located in putative promoter regions (1 kb upstream regions of TSSs). **b** The top enriched motif in the cold-specific DHSs located in exons. **c** The second most enriched motif in the cold-specific DHSs located in exons. **d** The top enriched motif in the cold-specific DHSs located in introns. The top 1000 cold-specific DHSs were selected based on read density ranking and were analyzed for regions of promoters, exons and introns, respectively

We scanned for DNA sequence motifs enriched in the top 1000 cold-specific DHSs (selected based on read density ranking) located in promoter regions and compared them to published transcription factor binding motifs [41]. A Dof (DNA-binding One Zinc Finger) transcription factor (TF) targeting motif was the most significantly enriched sequence found in 14.4% (144/1000) of the DHSs (*E* value = 3.0e−76) (Fig. 3). Dof TFs are members of a major plant-specific TF family that play important roles in development and response to multiple stresses, including cold, heat, and drought, and have been documented in several plant species [42]. We then scanned for DNA sequence motifs potentially enriched in cold-specific DHSs located in exons and introns. Interestingly, a GATA TF targeting motif was found to be the most significantly enriched sequence in exonic DHSs (*E* value = 7.2e−62), occupying 34% (342 of the top 1000 exonic DHSs) of the DHSs (Fig. 3). Plant-specific GATA TFs, such as ZML1 and ZML2, have been implicated in response to multiple environment stresses, including cold/freezing [43], excessive light [44], wound [45], and low nitrogen stresses [46]. Thus, potato GATA orthologs may play a regulatory role for genes (in tuber tissue) that respond to cold stress. The second most significantly enriched motif consisted of a repetitive 3mer (GAA) (*E* value = 2.1e−26), residing in 16.4% (164/1000) of the exonic DHSs (Fig. 3). This motif was previously identified as a PRC2-binding motif in both plants [47] and animals [48], suggesting that PRC2, which primarily catalyzes trimethylation of H3K27, may be recruited to these DHSs.

Searches among cold-specific DHSs located in introns revealed a mononucleotide G motif (Fig. 3), which was the most significantly enriched sequence (*E* value = 7.0e−86) detected in 62 of the top 1000 intronic DHSs. This motif overlaps with the binding motifs of MYB TFs (such as MYB55), which are known to be involved in multiple stress responses [49], including cold stress [50]. In addition, the consecutive G motif has also been demonstrated as a target substrate for PRC2 binding both in vivo and in vitro [51]. Taken together, cold-induced DHSs were enriched with DNA motifs targeted by TFs that are associated with plant stress responses and bound to PRC2 complex associated with H3K27 trimethylation.

### Elevated DNase I sensitivity associated with genes expressed in cold tubers

**Fig. 4:**
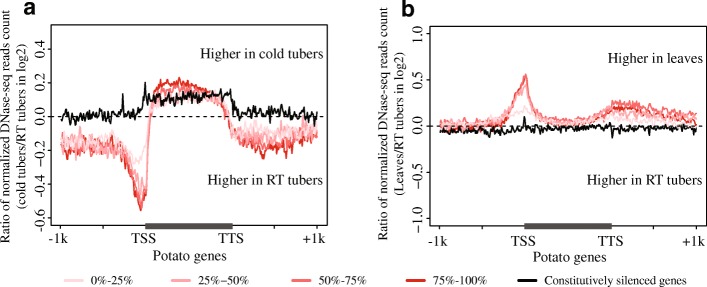
Dynamics of DNase I sensitivity between temperature treatments and between tissue types for genes in different expression level categories in potato. **a** Comparison of DNase I sensitivity for genes with different expression levels (FPKM) between cold tubers and RT tubers. **b** Comparison of DNase I sensitivity for genes with different expression levels between leaves and RT tubers. Active genes were identified if they were expressed in both temperature conditions with FPKM greater than 1 or in both tissues with FPKM greater than 1. Active genes were divided into 4 groups and sorted from low expression (0–25%) to high expression (75–100%) according to their expression levels (FPKM) in each sample. Constitutively silenced genes were genes with FPKM equal to 0 in all tissues and temperature treatments. Genes were divided into 100 bins and aligned together from TSSs to TTSs. Gene flanking regions (± 1 kb) were also analyzed in 100 bins

It is intriguing why so many DHSs emerged within gene body regions upon cold stress. We grouped genes into different bins based on expression level (FPKM) quartiles independently across tissues and treatments. Generally, highly expressed genes were characterized by higher DNase I sensitivity. The highest DNase I sensitivity was observed in regions 200 bp upstream of the TSSs (Additional file 1: Figure S6). We compared the levels of DNase I sensitivity for all genes between RT tubers and cold tubers (representative of cold stress). Genes expressed in both RT and cold tubers (FPKM > 1) showed a reduced level of DNase I sensitivity in 1 kb upstream and downstream regions of TSSs and TTSs, respectively, upon cold stress (Fig. 4a). Surprisingly, an elevation of DNase I sensitivity was detected across the gene body regions of expressed genes (FPKM > 1 in both RT and cold tubers) upon cold stress (Wilcoxon rank sum test, *p* < 2.2e−16) (Fig. 4a). More surprisingly, a similar trend was also observed across the gene body regions of constitutively silenced genes (FPKM = 0 in both RT and cold tubers) (Wilcoxon rank sum test, *p* < 2.2e−16) (Fig. 4a).

Similarly, we compared levels of DNase I sensitivity for all genes between leaves and RT tubers (representative of tissue specificity). Genes expressed in both leaves and RT tubers showed relatively higher levels of DNase I sensitivity in leaves for regions 200 bp upstream and downstream of TSSs and TTSs, respectively (Fig. 4b). However, most genes maintained similar DNase I sensitivity levels within their gene body regions between leaves and RT tubers (Fig. 4b). Constitutively silenced genes maintained a similar level of DNase I sensitivity throughout their gene body and flanking regions (Fig. 4b).

### Deposition of H3K27me3 to the gene body regions of active genes in cold tubers

**Fig. 5:**
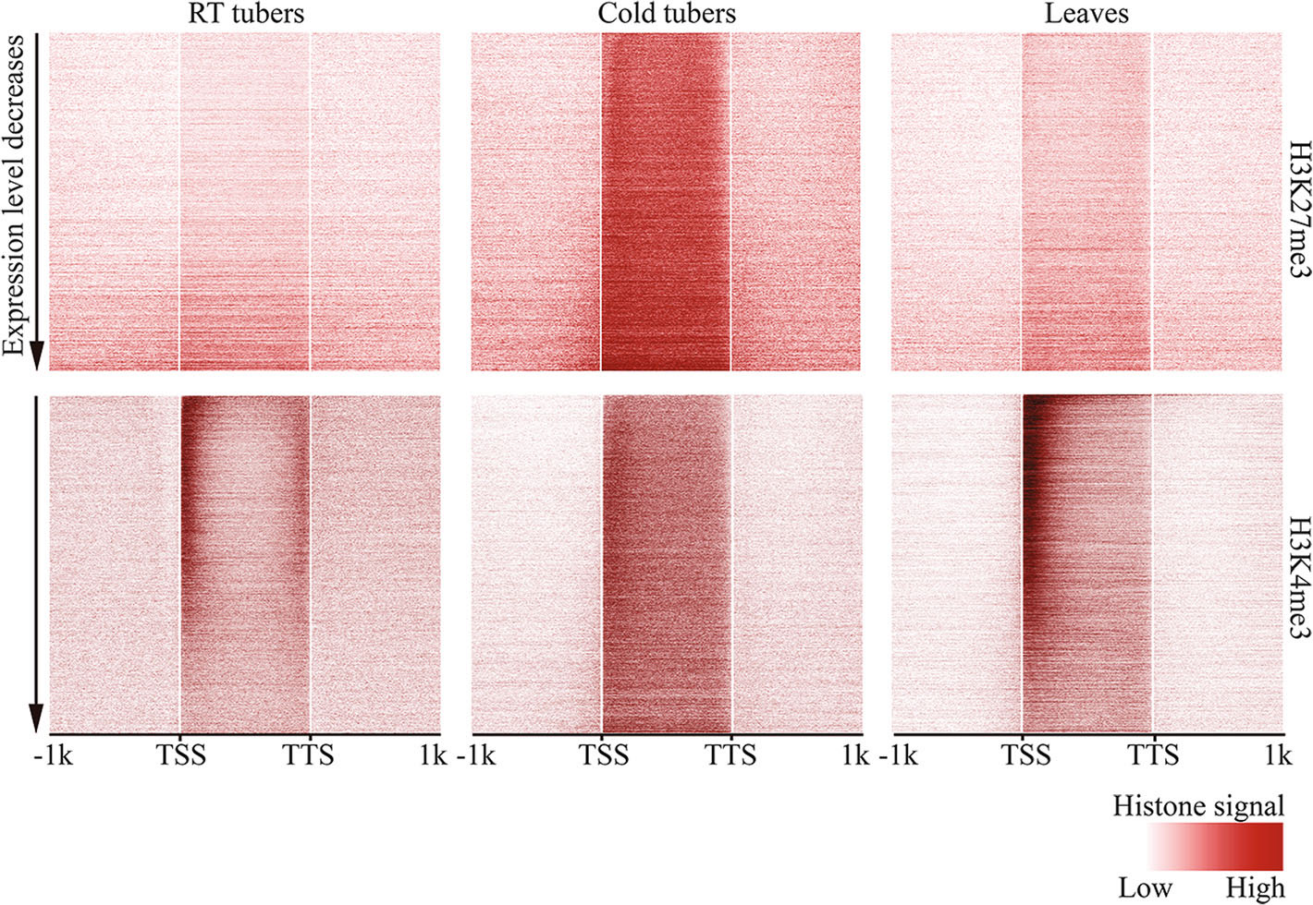
Heatmap of histone modifications surrounding active genes in RT tubers, cold tubers, and leaves of potato. Genes were sorted based on their expression levels with FPKM greater than 1 in each sample. Genes were divided into 100 bins and aligned together from TSSs to TTSs. Gene flanking regions (± 1 kb) were analyzed in 100 bins. Histone modification level was measured by quantifying the mid-point of pair-end ChIP-seq reads within each bin and normalizing by ChIP-seq read number per bp genome region per million mapped reads. Top panels: histone modification H3K27me3. Bottom panels: histone modification H3K4me3

Chromatin modifications, including histone modifications, play an integral role in plant responses to environmental stresses [16, 17]. We wondered if the distinct DHS pattern observed in cold tubers (Fig. 4a) was associated with any specific histone modifications. We conducted chromatin immunoprecipitation followed by sequencing (ChIP-seq) using antibodies against H3K27me3, a chromatin mark associated with repression of transcription [22, 52] (Additional file 2: Table S5). Interestingly, the gene body regions of expressed genes in cold tubers (FPKM > 1) were enriched with H3K27me3 (Fig. 5). Highly expressed genes in cold tubers were associated with relatively lower levels of H3K27me3 at the 5′ and 3′ ends (Fig. 5). This unique H3K27me3 enrichment was not observed with active genes in RT tubers or leaves (Fig. 5).

**Fig. 6:**
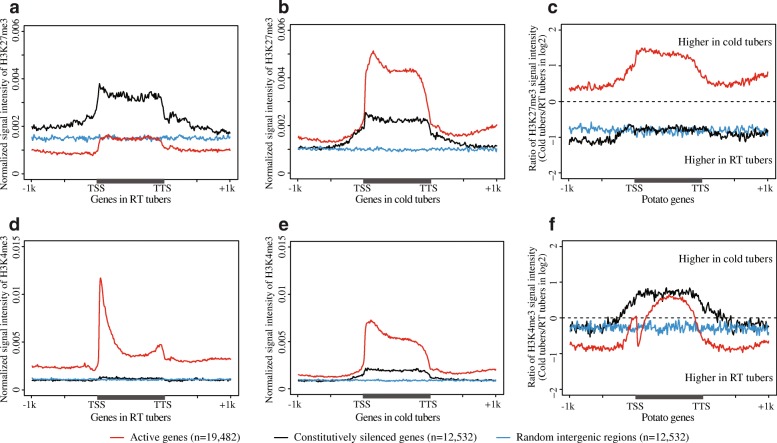
Histone modifications associated with potato genes. **a** H3K27me3 for active genes in RT tubers. **b** H3K27me3 for active genes in cold tubers. **c** The ratio of H3K27me3 signal intensity between cold vs. RT tubers. **d** H3K4me3 for active genes in RT tubers. **e** H3K4me3 for active genes in cold tubers. **f** The ratio of H3K4me3 signal intensity between cold vs. RT tubers. The same set of active genes (*n* = 19,482) and constitutively silenced genes (*n* = 12,532) were used in all analyses. Each active gene showed transcription (FPKM > 1) in both RT and cold tubers. Constitutively silenced genes did not show transcription in either RT or cold tubers (FPKM = 0). “Random intergenic regions” were randomly selected from regions that were at least 2 kb away from any annotated genes. The length and number of the random intergenic regions were the same as constitutively silenced genes. Genes were divided into 100 bins and aligned together from TSSs to TTSs. Genes flanking regions (± 1 kb) were analyzed in 100 bins. Histone modification signal was measured by quantifying the mid-point of pair-end ChIP-seq reads within each bin and normalizing by ChIP-seq read number per bp genome region per million mapped reads

To further characterize the distinct enrichment of H3K27me3 associated with actively expressed genes in cold tubers, we interrogated H3K27me3 levels for active genes (expressed in both RT and cold tubers; FPKM > 1; *n* = 19,482), constitutively silenced genes (not expressed in either RT or cold tubers; FPKM = 0; *n* = 12,532), as well as a set of random control sequences (intergenic sequences at least 2 kb away from any annotated genes; *n* = 12,532). In RT tubers, constitutively silenced genes were clearly associated with H3K27me3 (Fig. 6a). The average level of H3K27me3 associated with active genes was similar to intergenic sequences (Fig. 6a). In contrast, the level of H3K27me3 over active genes from cold tubers was significantly higher than constitutively silenced genes (Wilcoxon rank sum test, *p* < 2.2e−16) (Fig. 6b). Comparison of the H3K27me3 signals between RT and cold tubers revealed that only active genes displayed significant enrichment of H3K27me3 in cold tubers (Wilcoxon rank sum test, *p* < 2.2e−16) (Fig. 6c), while nucleosome density of these genes remains similar between RT and cold tubers (Additional file 1: Figure S7), suggesting the enrichment of H3K27me3 rather than enrichment of nucleosomes in cold tubers. In addition, regardless of the direction of differential expression (upregulated genes: *n* = 7096; downregulated genes: *n* = 6622; and constitutively expressed genes: *n* = 5764), all expressed genes upon cold stress displayed similarly increased enrichment of H3K27me3 (Additional file 1: Figure S8). Quantification of H3K27me3 signal intensity within gene body regions (from TSSs to TTSs) revealed that the levels of H3K27me3 were increased in 92% (17,913/19,482) of active genes upon cold stress (Wilcoxon rank sum test, *p* < 2.2e−16) (Additional file 1: Figure S9), whereas most constitutively silenced genes (79%, 9857/12,532) (Wilcoxon rank sum test, *p* < 2.2e−16) and intergenic regions (73%, 9114/12,532) (Wilcoxon rank sum test, *p* < 2.2e−16) displayed decreased levels of H3K27me3 (Additional file 1: Figure S9). These results collectively indicate that H3K27me3 is preferentially deposited to the gene body regions of active genes upon cold stress.

### A distinct H3K4me3 pattern associated with active genes in cold tubers

We then conducted ChIP-seq for H3K4me3, a mark associated with active transcription [53] (Additional file 2: Table S5). In RT tubers, H3K4me3, as expected, was only associated with actively transcribed genes (Fig. 6d). Consistent with observations in model animal and plant species [21, 54], H3K4me3 was most enriched at the 5′ end of active genes in RT tubers (Fig. 5, Fig. 6d) and leaves (Fig. 5). Strikingly, in cold tubers, H3K4me3 became more widely distributed toward the center of gene body regions of all active genes (Figs. 5 and 6e). Interestingly, we observed the deposition of H3K4me3 within constitutively silenced genes in cold tubers (Fig. 6e). Comparison of the H3K4me3 signals in RT versus cold tubers revealed increased levels of H3K4me3 in the gene body regions of both active and constitutively silenced genes (Wilcoxon rank sum test, *p* < 2.2e−16), as well as in the 5′ and 3′ regions of constitutively silenced genes in cold tubers (Wilcoxon rank sum test, *p* = 1.2e−08) (Fig. 6f).

We conducted ChIP-seq for one additional histone modification mark H4K5,8,12,16ac (Additional file 2: Table S5), a mark generally associated with transcription [55]. The distribution of H4K5,8,12,16ac along active genes was similar to patterns of H3K4me3 in RT tubers (Additional file 1: Figure S10a) and was enriched at both the 5′ end and gene body regions in cold tubers (Additional file 1: Figure S10b). However, unlike H3K4me3, we did not observe the deposition of this mark in constitutively silenced genes upon cold treatment (Additional file 1: Figure S10c).

### Bivalent H3K4me3-H3K27me3 mark associated with active genes in cold tubers

Coexistence of H3K4me3 and H3K27me3 on the same nucleosomes, known as bivalent histone marks, is well known to be associated with the promoters of poised genes responsible for differentiation and development in mammalian stem cells [28, 56]. In cold-stressed potato tubers, we observed the depositions of both H3K4me3 and H3K27me3 in actively expressed genes (FPKM > 1 in both RT and cold tubers) (Fig. 6). Both histone modification signal peaks oscillated with well-phased nucleosomes (Additional file 1: Figure S11a, b). The oscillating peaks derived from H3K4me3 and H3K27me3 signals in cold tubers nearly overlapped (Additional file 1: Figure S11b), suggesting that H3K4me3 and H3K27me3 may be deposited to the same nucleosomes upon cold stress.

**Fig. 7:**
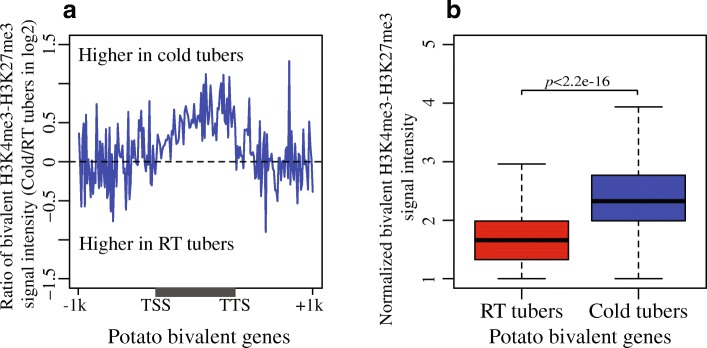
Profiles of bivalent H3K4me3-H3K27me3 histone modifications present in active genes in potato upon cold stress. **a** Active genes (*n* = 6442) in potato tubers were associated with enrichment of the bivalent H3K4me3-H3K27me3 mark in gene body regions upon cold stress. Genes were divided into 100 bins and aligned together from TSSs to TTS. Genes flanking regions (± 1 kb) were analyzed in 100 bins. Each active gene showed transcription (FPKM> 1) in both RT and cold samples. **b** Bivalent H3K4me3-H3K27me3 modification levels for the gene body regions of the active genes in the potato RT and cold tubers. Gene body regions indicate the genomic regions from TSSs to TTSs. Bivalent H3K4me3-H3K27me3 histone modification signal was measured by quantifying the mid-point of pair-end sequential K4-K27 ChIP-seq reads within each bin and normalized to control data K4-noAb and to the read number per bp genome region per million mapped reads

To examine whether the bivalent mark of H3K4me3 and H3K27me3 were truly deposited on the same nucleosomes, sequential ChIP [32] was conducted by first ChIP using an anti-H3K4me3 antibody followed by second ChIP using an anti-H3K27me3 antibody (designated as K4-K27). A control sequential ChIP was also performed with no antibody included in the second ChIP, which would eliminate the possibility that the enrichment was due to carry-over from the first antibody (anti-H3K4me3). In addition, we performed sequential ChIP using antibodies in the reverse order (first anti-H3K27me3 and second anti-H3K4me3, designated as K27-K4; first anti-H3K27me3 and no antibody as control) (see the “Methods” section) (Additional file 2: Table S5). We evaluated the sequential ChIP data by selecting two groups of expressed genes (FPKM > 1) in cold tubers that associated only with H3K4me3 or H3K27me3, respectively, as well as an additional group of expressed genes (FPKM > 1) that associated with both H3K4me3 and H3K27me3. Bivalent histone modifications were only detected for genes that are associated with both histone marks (Additional file 1: Figure S12). We only retained high confident genes for further analyses: these genes were associated with enrichment of bivalent mark across the gene body regions in both K4-K27 and K27-K4 sequential ChIP-seq data, upon cold stress. Remarkably, we found that 33% (6442/19,482) of the active genes displayed enrichment of true bivalent mark on the same nucleosomes upon cold stress (Fig. 7a, b).

Bivalent H3K4me3-H3K27me3 domains were previously found to be associated with the histone modification H3K4me1 in human CD4^+^ memory T cells [57]. Therefore, we also conducted H3K4me1 ChIP-seq to examine a potential association of this mark with bivalent mark-associated genes in cold tubers. Interestingly, we found that H3K4me1 was exclusively enriched in the bivalent mark-associated genes (*n* = 6442) upon cold stress (Additional file 1: Figure S13a), whereas the remaining active genes (*n* = 13,040) were associated with decreased levels of H3K4me1 (Additional file 1: Figure S13b). Thus, the bivalent H3K4me3-H3K27me3 mark in cold tubers showed a similar pattern of association with H3K4me1 as previously observed in human CD4^+^ memory T cells.

### Expression of genes associated with bivalent H3K4me3-H3K27me3 mark

We analyzed the expression of the 6442 genes associated with bivalent H3K4me3-H3K27me3 mark in cold tubers. Unlike the proposed repressive effect of the bivalent H3K4me3-H3K27me3 mark in mammalian species [30, 58], we did not observe an overall decrease in expression of these genes in cold tubers. Instead, we found that 3064 (47.5%) genes were upregulated upon cold stress, while 1994 (31%) genes were downregulated, and 1384 (21.5%) genes were constitutively expressed (expression level not significantly changed). In addition, these three groups of genes showed a similar level of bivalent mark signal intensity in cold tubers (Additional file 1: Figure S14a), whereas their expression levels were significantly different (Wilcoxon rank sum test, *p* < 2.2e−16) (Additional file 1: Figure S14b). These results suggest that the level of the bivalent histone modifications tends to be independent of the direction and extent of gene expression level changes.

**Fig. 8:**
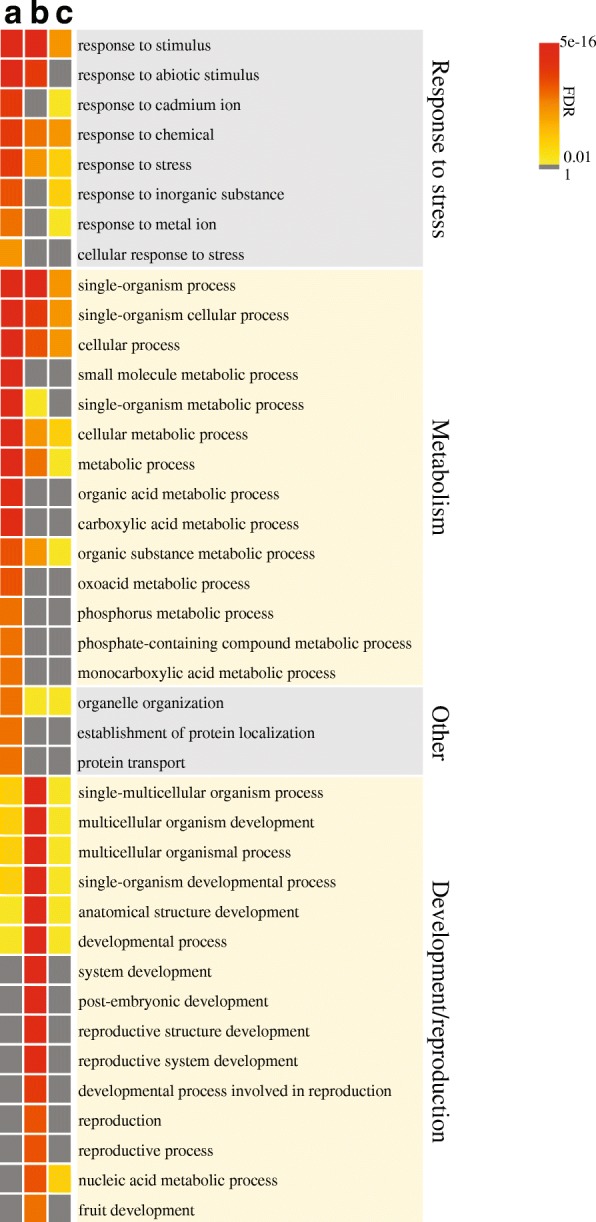
Heatmap of FDR for Gene Ontology (GO) terms enriched for the bivalent mark-associated genes in potato tubers upon cold stress. **a** Upregulated genes associated with the bivalent mark. **b** Downregulated genes associated with the bivalent mark. **c** Constitutively expressed genes associated with the bivalent mark

Next, we investigated the functional annotations of the bivalent mark-associated genes by identifying the closest *A. thaliana* homologs of the potato genes and scanning for enriched Gene Ontology (GO) terms. The bivalent mark-associated genes, especially the upregulated genes, were mainly associated with functions related to stress response and metabolic processes (Fig. 8). Strikingly, the GO terms for developmental and reproductive processes were exclusively associated with downregulated genes with the bivalent mark (Fig. 8), which is similar to the reports that bivalent H3K4me3-H3K27me3 mark is thought to repress developmentally related genes in embryonic stem cells (ESCs) and sperm but poise them for rapid activation [59, 60].

### Chromatin accessibility of the bivalent mark-associated genes

To further examine if the genes associated with the bivalent H3K4me3-H3K27me3 mark tend to be more accessible, we compared the DNase I sensitivity levels in the genic regions between the bivalent mark-associated genes and the remaining active genes. In cold tubers, the underlying chromatin of upregulated and constitutively expressed genes with bivalent marks were significantly more accessible (Wilcoxon rank sum test, upregulated genes, *p* = 5.49e−09, constitutively expressed genes *p* = 7.02e−11) compared to the remaining upregulated and constitutively expressed genes, respectively. Downregulated genes with bivalent marks showed a similar level of the DNase I sensitivity compared to the remaining downregulated genes (Wilcoxon rank sum test, *p* = 0.34) (Additional file 1: Figure S15a-c). In addition, we compared the levels of DNase I sensitivity change between cold tubers and RT tubers for both gene sets and observed a greater elevation in DNase I sensitivity in bivalent mark-associated genes upon cold stress, including upregulated (Wilcoxon rank sum test, *p* = 5.07e−07), downregulated (Wilcoxon Rank Sum test, *p* = 1.37e−05), and constitutively expressed genes (Wilcoxon Rank Sum test, *p* = 0.03) (Additional file 1: Figure S15d-f). These results suggest that the chromatin of bivalent mark-associated genes becomes more accessible upon cold stress. Interestingly, downregulated and constitutively expressed genes with bivalent marks displayed less fold changes in expression compared to the remaining downregulated and constitutively expressed genes, respectively, upon cold stress (Additional file 1: Figure S15 g-i), indicating the greater elevation in DNase I sensitivity in bivalent mark-associated genes seems to be not related to greater fold changes in transcription. These results suggest that the bivalent histone modifications may foster a chromatin environment with greater accessibility as a response to cold stress.

## Discussion

Chromatin domains associated with the simultaneous presence of both H3K4me3 and H3K27me3 on the same nucleosomes were first described as bivalent domains in mouse ESCs [28, 29]. Such bivalent domains mostly overlap with promoters of genes that encode TFs involved in the development and lineage specificity [28, 58]. In ESCs, lineage-specific genes associated with the H3K4me3-H3K27me3 bivalent domains are not expressed at significant levels in order to maintain pluripotency [28, 61]. However, upon differentiation, the majority of these lineage-specific genes will lose the bivalent domains [28]. Some lineage-specific genes become activated accompanying with loss of H3K27me3, whereas some become silenced with loss of H3K4me3 but retaining of H3K27me3 [58]. In bivalent domains, the H3K4me3 associated with inactive genes in ESCs is postulated to facilitate future activation of these genes by assisting the recruitment of RNA polymerase II, while H3K27me3 is postulated to repress developmental genes by preventing RNA polymerase II from accumulating at promoters and from the release of paused phase into the elongation phase [30]. Therefore, bivalent domains are thought to poise genes for activation while keeping them repressed.

We observed a unique bivalent H3K4me3-H3K27me3 mark deposited to a large number (6442) of active genes in the cold-stressed potato tubers (Fig. 7a, b). In addition, we found that H3K4me1 was exclusively enriched in the bivalent mark-associated genes in cold tubers, which resembles the association of the same bivalent mark with H3K4me1 reported in mammalian species [57], suggesting that the cold-induced bivalent H3K4me3-H3K27me3 mark may play a specific role in responses to cold stress. It will be interesting to examine if this bivalent mark is associated with plant response to other abiotic stresses.

In animals, most genes associated with H3K4me3-H3K27me3 bivalent domains were partially repressed [28, 29]. We found that only a subset of potato genes associated with the same bivalent mark in cold tubers were downregulated upon cold stress. Interestingly, these genes were mostly related to developmental and reproductive processes (Fig. 8), suggesting the bivalent mark may play a similar repressive role on developmental genes. In contrast, we found that bivalent mark-associated genes were also involved in stress response and metabolic processes (Fig. 8) and many of these genes were upregulated upon cold stress. This upregulation is correlated with the fact that the cold-specific DHSs are enriched with several DNA motifs bound to TF families that play a role in stress responses (Fig. 3). Upregulation of transcription was previously found to be associated with several dehydration response memory genes in *Arabidopsis*. Interestingly, these genes were reported to be associated with both H3K4me3 and H3K27me3 marks [33], although it is unclear if these previously reported H3K4me3 and H3K27me3 marks were associated with the same nucleosomes. Thus, the cold stress-induced bivalent domains may also play a role to promote the transcription of genes involved in the cold response. The deposition of H3K27me3 at bivalent promoters was originally hypothesized to repress lineage regulatory genes and to maintain the pluripotent state of ES cells [28, 30]. However, recent studies suggest that the deposition of H3K27me3 does not prevent genes from transcription. For example, in mouse rib chondrocytes, approximately 89% of genes enriched with H3K27me3 were not sufficiently repressed [62]. In *Arabidopsis*, the dehydration stress memory genes associated with high levels of H3K27me3 were not repressed from transcription activation during dehydration stress [33]. One possible explanation for the diverse functions of H3K27me3 is that H3K27me3 could interact with a polycomb-group (PcG) protein, LHP1 (LIKE HETEROCHROMATIN PROTEIN1), which plays dual roles as an activator and a repressor of gene expression in a context-dependent manner [63, 64]. Interestingly, we found that putative potato *LHP1* ortholog was significantly upregulated in the cold-stressed tubers, detected by both RNA-seq (Additional file 2: Table S6) and qRT-PCR (*t* test, *p* < 0.01) (Additional file 1: Figure S16a). Besides, the putative CLF, a major methyltransferase for H3K27me3 in plants [65, 66], as well as another two cold-inducible PcG components MSI1 (MULTICOPY SUPRESSOR OF IRA 1) [63] and VEL1 (VIL2/VERNALIZATION5/VIN3-LIKE1) [67] was also significantly upregulated in cold tubers (Additional file 2: Table S6, Additional file 1: Figure S16a), which suggests the potato CLF ortholog and other PcG components may play a role in regulation of cold-induced deposition of H3K27me3 in potato tubers. Therefore, we postulate that H3K27me3 in the cold-induced bivalent domains may grant a specific chromatin environment that may facilitate either upregulation or downregulation of genes.

The cold-induced bivalent mark in the potato tubers notably spread across gene body regions (Fig. 7a), whereas the bivalent mark in mammalian ESCs is mainly enriched in promoter regions [28]. Intriguingly, the distribution of the bivalent mark in gene body regions (Fig. 7a) coincided with the elevation of DNase I sensitivity in the same regions (Fig. 4a) in potato tubers. Furthermore, we observed a greater elevation in chromatin accessibility for the bivalent mark-associated genes compared to the remaining active genes (Additional file 1: Figure S15d-f). Thus, chromatin domains associated with the bivalent mark become more accessible, possibly to the access of regulatory proteins, upon cold stress. It was postulated that the lineage-specific genes associated with the bivalent mark are transcriptionally poised by H3K4me3 in ESCs [28]. However, evidence for this poising model is sparse [30]. Study in mouse ESCs suggests that H3K4me3 is strongly correlated with the binding of a chromatin remodeler, Chromodomain Helicase DNA-Binding protein 1 (CHD1), which is required for maintenance of open chromatin and pluripotency of ESCs [68]. Here, we observed the cold-induced deposition of H3K4me3 in active genes, which is coincident with the significant upregulation of a major methyltransferase *ATXR3* [31, 69] and a few “readers” for H3K4me3 in cold tubers, based on either RNA-seq (Additional file 2: Table S7) or qRT-PCR (Additional file 1: Figure S16b). The enrichment of H3K4me3 in cold tubers is well correlated with the increases in DNase I sensitivity for active genes (Pearson’s correlation *r* = 0.77, *p* < 2.2e−16) upon cold stress (Figs. 4a and 6f). In addition, we observed a greater level of H3K4me3 enrichment in the bivalent mark-associated genes (Additional file 1: Figure S17). Such a correlation between H3K4me3 in bivalent domains and chromatin accessibility is supported by a recent study in mESC that loss of MLL2 (histone methyltransferase for H3K4me3) resulted in significantly decreased levels of chromatin accessibility as well as H3K4me3 at bivalent promoters [70]. Therefore, we postulate that H3K4me3 in the cold-induced bivalent domains may involve in assisting the maintenance of chromatin accessibility.

**Fig. 9:**
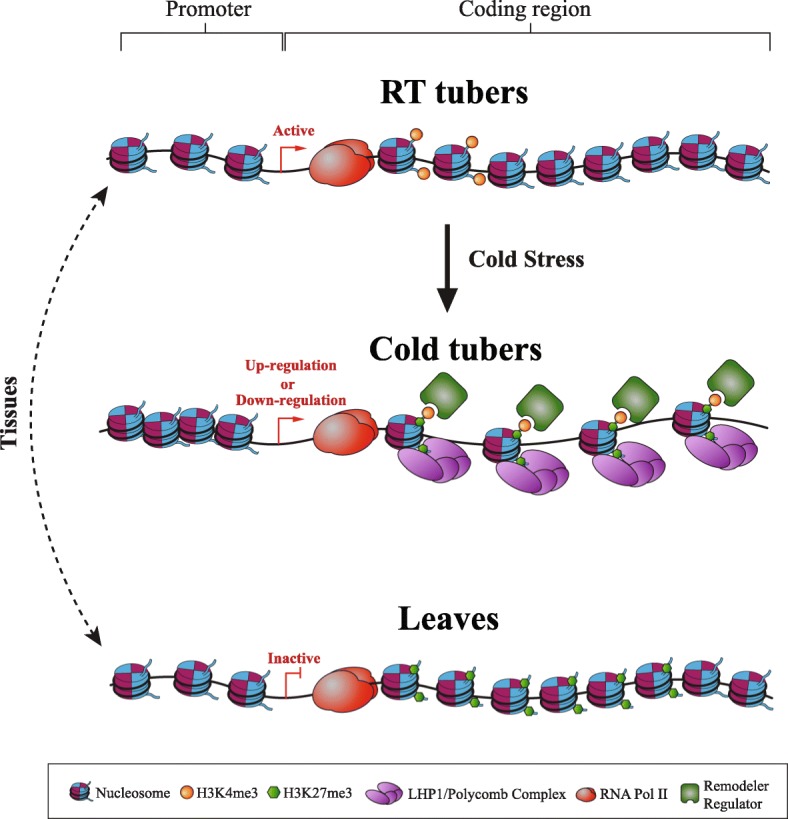
A model of chromatin dynamics associated with genes in potato tubers under cold stress. In ambient condition, active genes in RT tubers maintain open chromatin in their putative promoters and H3K4me3 at their 5′ ends, while the gene bodies are less accessible to DNase I and are depleted of H3K27me3. Some of these genes could be active in leaves as well and maintain similar chromatin features. However, other genes could be inactive in leaves and switch to a repressive chromatin state in which the gene bodies are marked by H3K27me3 and are depleted of H3K4me3. In contrast, cold stress in tubers can result in upregulation of a set of stress-response genes as well as downregulation of a set of developmental genes. This regulation of transcription is associated with the deposition of the bivalent H3K4me3-H3K27me3 mark. Existence of H3K4me3 across gene body regions may ensure the chromatin accessibility through chromatin remodelers and regulators, while the spread of H3K27me3 may provide a chromatin environment for either gene activation or repression via LHP1. Thus, the bivalent H3K4me3-H3K27me3 mark represents a distinct chromatin environment with greater accessibility, which may facilitate the access of regulatory proteins required for gene upregulation or downregulation in response to the cold stress

We propose a model of chromatin dynamics associated with gene expression in potato tubers under cold stress (Fig. 9). In ambient condition, active genes in RT tubers maintain open chromatin in their putative promoters and H3K4me3 at their 5′ ends, while the gene bodies are less accessible to DNase I and are depleted of H3K27me3. Some of these genes could be active in leaves as well and maintain similar chromatin features (not shown in the model). However, the other genes could be inactive in leaves and switch to a repressive chromatin state in which the gene bodies are marked by H3K27me3 and are depleted of H3K4me3 (Fig. 9). In contrast, cold stress in tubers can result in upregulation of a set of stress-response genes as well as downregulation of a set of developmental genes. This regulation of transcription is associated with the deposition of the bivalent H3K4me3-H3K27me3 mark (Fig. 9). Existence of H3K4me3 across gene body regions may ensure the chromatin accessibility through chromatin remodelers and regulators, while the spread of H3K27me3 may provide a chromatin environment for either gene activation or repression via LHP1. Thus, the bivalent H3K4me3-H3K27me3 mark represents a distinct chromatin environment with greater accessibility, which may facilitate the access of regulatory proteins required for gene upregulation or downregulation in response to the cold stress. Although the bivalent mark-associated genes were found to be differentially expressed in relation to various biological function, further experimentation will be necessary to establish a mechanistic role for the bivalent mark in the regulation of gene expression.

## Conclusions

The cold stress induced a large number of DHSs that were enriched in genic regions (exons and introns), which tend to be frequently associated with differential gene expression in response to temperature variation. The cold stress also induced enhanced chromatin accessibility for active genes upon cold stress. A large number of active genes (6442) in cold-stored tubers were associated with bivalent histone modifications of H3K4me3 and H3K27me3 in their body regions. The bivalent mark-associated genes were more accessible than other genes, while the direction of their expression upon cold stress were generally associated with their biological functions. We hypothesize that the bivalent H3K4me3-H3K27me3 mark represents a distinct chromatin environment with greater accessibility, which may facilitate the access of regulatory proteins required for gene upregulation or downregulation in response to cold stress in potato tubers.

## Methods

### Plant materials

The doubled monoploid potato clone DM1-3 516 R44 (DM, a homozygous line, *S. tuberosum* Group Phureja, 2*n* = 2*x* = 24) was used for this study and is the reference genotype for potato [38, 71]. DM plants were grown in a greenhouse with the photoperiod of 16-h daylight at 22 °C and 8-h darkness at 16 °C. Leaves and tubers were collected simultaneously from the same plants 70 days after planting. Leaves were ground into fine powder in the liquid nitrogen immediately after harvesting. Tubers collected from a single plant were divided into two groups. Each group was treated at room temperature (22 °C) and cold temperature (4 °C) for 14 days, respectively, and then immediately ground into fine powder in the liquid nitrogen. Powder samples were stored at − 80 °C for experiments in this study. We chose to treat potato tubers under 4 °C for 14 days to simulate the condition for potato Cold-induced sweetening (CIS), since the key gene *Vacuolar Invertase* (*VInv*), which plays a critical role in CIS in potato tubers, is significantly upregulated and reaches to the peak after approximately 14 days [35].

### DNase-seq, RNA-seq, and ChIP-seq

Two biological replicates of DNase-seq libraries were developed from each tissue or treatment. Nuclei extraction, DNase I digestion, and DNase-seq library construction followed published protocols [72]. Briefly, 10 g of finely ground powder was suspended in the same volume of pre-chilled nuclear isolation buffer (NIB; 10 mM Tris-HCl, 80 mM KCl, 10 mM EDTA, 1 mM spermidine, 1 mM spermine, 0.15% mercaptoethanol, 0.5 M sucrose, pH 9.5) as the volume of powder for nuclei isolation. The prepared nuclei pellet was suspended in nuclear digestion buffer (NDB; 10 mM Tris-HCl, 10 mM NaCl, 3 mM MgCl_2_, pH 7.4) for DNase I digestion. The digestion was conducted with gradient concentrations (0.04–4 units) of DNase I for 10 min at 37 °C. Digestion patterns were visualized and assessed using a Pulsed-Field Gel Electrophoresis (PFGE) system (Bio-Rad, Cat.# 170-3615) with the program of 20–60 s switch time for 17.5 h at 6 V/cm. The running process was performed in a cold room (10 °C). After DNase I digestion, high molecular weight (HMW) DNA was isolated and blunt end withT4 DNA polymerase (NEB, Cat. #0203 L). HMW DNA was then ligated with adapter I (5′-Biotin-ACAGGTTCAGAGTTCTACAGTCCGAC-3′ and 5′ P-GTCGGACTGTAGAACTCTGAAC-3′) and digested with MmeI. Restriction enzyme MmeI-treated ends were ligated with adapter II (5′-P-TCGTATGCCGTCTTCTGCTTG-3′ and 5′-CAAGCAGAAGACGGCATACGANN-3′). The adapter-ligated DNA was enriched using PCR with linker-specific primers (5′-CAAGCAGAAGACGGCATACGA-3 and 5′-AATGATACGGCGACCACCGACAGGTTCAGAGTTCTACAGTCCGA-3′). DNA fragments ~ 90 bp in length purified by PAGE were submitted for sequencing using an Illumina HiSeq platform in the single-end mode with 50-nucleotide reads.

Each replicate sample used for DNase-seq was prepared for RNA-seq. High-quality RNA was extracted using a RNeasy Plant Mini Kit (Qiagen, Cat. # 74904), followed by DNase I treatment to remove genomic DNA. About 5 μg of total RNA was converted to cDNA using the TruSeq mRNA-seq kit from Illumina, and multiplexed cDNA libraries were sequenced on an Illumina HiSeq platform in the single-end mode, generating 50-nucleotide reads.

Chromatin immunoprecipitation followed by sequencing (ChIP-seq) was performed following published protocols [73], using the same samples that were used for DNase-seq. Antibodies against H3K27me3 (Millipore 07-449), H3K4me3 (Abcam 8580), H3K4me1 (Abcam 8895), and H4K5,8,12,16 ac (Millipore 06-598) were used in ChIP experiments. ChIP-seq libraries for Illumina sequencing were constructed according to the protocol of “preparing samples for ChIP sequencing of DNA” provided by Illumina. Briefly, extracted nuclei were digested into monomer nucleosome pattern (~ 150 bp fragments) using MNase (Sigma N3755). Target chromatin fragments were captured using corresponding antibodies and precipitated with rProtein A sepharose (GE 17-1279-01). ChIPed DNA was extracted from precipitated chromatin for ChIP-seq library preparation. ChIPed DNA was end-repaired using an End-It DNA end repair kit (Epicenter, ER0720). The “dA” base was then added to 3′ ends of the end-repaired DNA fragments using Klenow fragment (NEB, M0212S), followed by Illumina adapter ligation for pair-end sequencing, using a quick ligase (NEB M2200). Adapter-ligated DNA fragments were purified by running a 2% agarose gel in TAE buffer and were size-selected from 150 to 300 bp. Purified adapter-ligated ChIPed DNA was enriched by 13 PCR cycles and purified by running a 2% gel for isolating DNA fragments in the range of 200–300 bp. Purified ChIP-seq libraries were sequenced on an Illumina HiSeq platform in either single-end or paired-end mode with 100- or 150-nucleotide reads.

### Sequential ChIP-seq

Sequential ChIP-seq was conducted using a Re-ChIP-IT kit (Active Motif, Cat # 53016) following the manufacturer’s instruction. The material that was used for DNase-seq was also used for sequential ChIP-seq. Potato tubers were sliced and then cross-linked with 1% formaldehyde for 10 min by vacuum infiltration. Cross-linked tissue was quickly quenched in 0.125 M glycine followed by 3 times of wash using ddH_2_O. After grinding, nuclei were isolated in nuclei extraction buffer (10 mM Tris-HCl pH 8.0, 0.25 M sucrose, 10 mM MgCl_2_, 1% Triton X-100, and protease inhibitors) and pelleted by centrifugation. Nuclei were re-suspended in buffer (50 mM Tris-HCl, pH 8.0, 10 mM EDTA, and 1% SDS) and fragmentized to 200–500 bp with an average size of ~ 300 bp using sonication (Qsonica Q700) for 2 min with settings of pulse on for 15 s and pulse off for 60 s on ice. Following the published strategy from *Arabidopsis* [32], chromatin was immunoprecipitated with anti-H3K4me3 and subsequent anti-H3K27me3, as well as anti-H3K27me3 and subsequent anti-H3K4me3, respectively, for each sample. In addition, chromatin from each sample was also immunoprecipitated with first antibody and followed by no antibody as control, to eliminate the possibility that the final enrichment was due to carry-over from the first antibody. Immunoprecipitated DNA was de-cross-linked and extracted for Illumina sequencing library construction. The library construction is the same as for regular ChIP-seq library. Sequential ChIP-seq libraries were sequenced on an Illumina HiSeq platform in paired-end mode with 150-nucleotide reads.

### Reverse transcription and quantitative real time-PCR

Reverse transcription was performed using Invitrogen SuperScript™ III Reverse Transcriptase kit (Invitrogen, Cat # 18080044) with oligo(dT)_20_ primer. The amounts of individual genes were measured by using gene-specific primers with SYBR Advantage qPCR Premix (Takara, Cat # 639676). Quantitative real-time-PCR (qRT-PCR) was conducted using a RT-PCR cycler (CFX connect Bio-Rad) with settings of initial denaturation 95 °C for 30 s, and 40 cycles of 95 °C for 15 s, 56 °C for 20 s, and 72 °C for 15 s. Three biological replicates from each treatment were used for quantifying relative expression for each gene. The expression of individual genes was normalized to the reference genes *EF1α* using the 2^−ΔΔCt^ calculation. Statistical significance was evaluated using *t* test. The specific primers used for potato genes are shown in Additional file 2: Table S8.

### Data analysis

The raw reads generated from DNase-seq, RNA-seq, ChIP-seq, and sequential ChIP-seq were processed for quality control using FASTQC program (http://www.bioinformatics.babraham.ac.uk/projects/fastqc). Reads were cleaned using Cutadapt v1.9.1 [74] with a minimum base quality of 20. Cleaned DNase-seq, ChIP-seq, and sequential ChIP-seq reads were aligned to the DM potato genome assembly (PGSC v4.04 [38]) using Bowtie 1 [75] with no mismatches allowed. Only reads that mapped to unique positions were used for further analysis.

DNase I hypersensitive sites (DHSs) were identified using in-house developed program Popera [76] (https://github.com/forrestzhang/Popera) with FDR < 0.05. Popera applies the kernel density estimation algorithm for the DHS identification, which is similar to the algorithm defined in F-seq [10]. DHSs were identified independently in each biological replicate for each sample. Overlapping DHSs (at least 1 bp overlap) between 2 biological replicates of a sample were retained for downstream analyses. The distribution of DNase-seq reads in the potato genome was revealed by calculating the coverage of unique reads (mapped to a unique genomic position) in each 100 bp non-overlapping window from the entire genome. The most frequent DNase I cutting site (a single base pair position) within a DHS was indicated from the DHS peak point calculated using the number of uniquely aligned DNase-seq reads. Genomic distribution of DHSs relative to annotated genes was determined if the most frequent DNase I cutting site within a DHS is located in a genomic feature. Tissue-specific and temperature-specific DHSs were identified if a DHS does not overlap (no single base pair overlap) any DHSs found in the other sample. To define the DNase I sensitivity, genes were aligned from transcription start sites (TSSs) to transcription termination sites (TTSs) and divided into 100 bins, while gene flanking regions were also partitioned into the same number of windows as genes. Normalized DNase-seq reads were plotted over aligned genes as well as their ± 1 kb flanking regions.

To determine the histone modification distribution from ChIP-seq and sequential ChIP-seq data, the mid-point of the uniquely aligned pair-end reads was set as the modification signal. The level for individual histone modification was measured by quantifying histone modification signals within an interval and normalizing to length of the interval, read number per base genome per million mapped reads, and input data. Similarly, the level of bivalent histone modifications was quantified within an interval using bivalent histone modification signals generated from sequential ChIP-seq with anti-H3K4me3 followed by anti-H3K27me3 (named K4-K27) and normalized to control sequential ChIP-seq with anti-H3K4me3 followed by no antibody (K4-noAb), the length of the interval, and read number per base genome per million mapped reads. The bivalent histone modification level was also measured for the same sample using sequential ChIP-seq with reversed order of antibodies (K27-K4 normalized to K27-noAb). Genes were processed for further analyses only if they displayed increased levels of bivalent histone modifications in both sequential ChIP-seq K4-K27 and K27-K4 data upon cold storage.

RNA-seq reads processed from quality control were mapped to the potato (PGSC v4.04 [38]) genome assemblies, using Tophat (v2.1.1) [77]. Cufflinks (v2.2.1) [78] was used to call the expression value (FPKM) of annotated potato genes. Differentially expressed genes were called using Cuffdiff (v2.21) and DEseq2 (v1.10.1) [79] with FDR < 0.01, respectively. Differentially expressed genes were used for further analyses if they were detected by both Cuffdiff and DEseq2. Similarly, genes were considered not differentially expressed or constitutively silenced if they were detected by both Cuffdiff and DESeq2. The programs of data process and statistical test were written and conducted in Perl or R (https://www.r-project.org). *z* test was conducted using two-tailed probability.

### Motif search

Cold-specific genic DHSs were split into those overlapping putative promoters, exons, and introns. The top 1000 DHSs based on peak read depths were used for further analysis. Motif scanning was conducted using meme-chip from the MEME suite tools [80]. Negative control sequences for cold-specific DHSs at promoters were constructed by taking the top 1000 promoter DHSs that overlapped in both RT and cold tubers data sets (shared promoter DHSs). Negative control sequences for cold-specific exonic and intronic DHSs were assembled similarly as cold-specific DHSs at promoters, except using exonic and intronic DHSs in lieu of promoter DHSs (shared exonic DHSs and intronic DHSs), respectively. All DHSs were aligned by their peak coordinates and scanned for motifs using 100 bp surrounding the peak coordinates.

### Gene ontology enrichment

A total of 6442 potato genes associated with bivalent H3K4me3-H3K27me3 mark upon cold stress were divided into three groups based on differential expression upon cold stress. Homologous sequences in *Arabidopsis thaliana* of the upregulated (*n* = 3064), downregulated (*n* = 1994), and constitutively expressed (*n* = 1384) potato bivalent mark-associated genes were identified, respectively, using the Blastp program (BLAST v2.2.31). The *Arabidopsis* homologous protein sequences with the highest similarity to the potato bivalent mark-associated genes were screened for enriched Gene Ontology terms using agriGO [81]. Enrichment test was conducted using Fisher’s exact test and the Benjamini–Hochberg FDR *P* value normalization. Background terms were set to all annotated *Arabidopsis* genes for each enrichment test.

## Additional files

Additional file 1: **Figure S1.** DNase-seq data correlation between two biological replicates derived from RT tubers, cold tubers, and leaves. **Figure S2.** Distribution of DHSs on all potato chromosomes. **Figure S3.** Distribution of DHSs derived from potato leaves. **Figure S4.** Comparison in number of temperature-specific genic DHSs between RT and cold tubers. **Figure S5.** RNA-seq data correlations between two biological replicates derived from RT tubers, cold tubers, and leaves. **Figure S6.** Relationship between DNase I sensitivity and gene expression levels in potato. **Figure S7.** The nucleosome density associated with potato genes. **Figure S8.** Profiles of histone modifications associated with potato genes upon cold stress. **Figure S9.** H3K27me3 levels of potato genes between RT and cold tubers. **Figure S10.** Profiles of histone modification H4K5, 8, 12, 16ac associated with potato genes. **Figure S11.** Histone modifications associated with active genes in cold-stressed tubers. **Figure S12.** Profiles of histone modifications associated with potato genes in cold tubers. **Figure S13.** Histone modification H3K4me1 levels of the potato bivalent mark-associated genes. **Figure S14.** Bivalent histone modification levels and expression levels of the potato bivalent mark-associated genes in cold tubers. **Figure S15.** DNase I sensitivity of the bivalent mark-associated genes in potato tubers upon cold stress. **Figure S16.** Expression levels of putative PcG and TrxG genes in potato tubers upon cold stress. **Figure S17.** Histone modification H3K4me3 associated with the bivalent mark-associated genes in potato tubers upon cold stress. (PDF 1782 kb)

Additional file 2: **Table S1.** DNase-seq data generated from potato RT tubers, cold tubers, and leaves. **Table S2.** RNA-seq data generated from potato RT tubers, cold tubers, and leaves. **Table S3.** Differentially expressed genes involved in potato tuber carbohydrate pathway under cold stress. **Table S4.** Differentially expressed genes involved photosynthesis in potato. **Table S5.** ChIP-seq and sequential ChIP-seq data generated from potato DM. **Table S6.** Expression of the genes associated with trimethylation of H3K27 in RT and 14-day cold-treated potato tubers. **Table S7.** Expression of the genes associated with trimethylation of H3K4 in RT and 14-day cold-treated potato tubers. **Table S8.** Primers used for qRT-PCR. (PDF 201 kb)

Additional file 3: Review history. (DOCX 1242 kb)

## Data Availability

All sequencing reads of DNase-seq, RNA-seq, and ChIP-seq generated from the potato DM during the current study are available from National Center for Biotechnology Information (NCBI) Sequence Read Archive (SRA) under accession number PRJNA373998 (https://www.ncbi.nlm.nih.gov/bioproject/PRJNA373998) [82].
