# Supplementary material for: Cold stress induces enhanced chromatin accessibility and bivalent histone modifications H3K4me3 and H3K27me3 of active genes in potato
Source: Genome Biol. 2019 Jun 17;20:123. doi: 10.1186/s13059-019-1731-2 (PMC6580510; doi:10.1186/s13059-019-1731-2)
Supplement: Supplementary file 1 — Figure S1. DNase-seq data correlation between two biological replicates derived from RT tubers, cold tubers, and leaves. Figure S2. Distribution of DHSs on all potato chromosomes. Figure S3. Distribution of DHSs derived from potato leaves. Figure S4. Comparison in number of temperature-specific genic DHSs between RT and cold tubers. Figure S5. RNA-seq data correlations between two biological replicates derived from RT tubers, cold tubers, and leaves. Figure S6. Relationship between DNase I sensitivity and gene expression levels in potato. Figure S7. The nucleosome density associated with potato genes. Figure S8. Profiles of histone modifications associated with potato genes upon cold stress. Figure S9. H3K27me3 levels of potato genes between RT and cold tubers. Figure S10. Profiles of histone modification H4K5, 8, 12, 16ac associated with potato genes. Figure S11. Histone modifications associated with active genes in cold-stressed tubers. Figure S12. Profiles of histone modifications associated with potato genes in cold tubers. Figure S13. Histone modification H3K4me1 levels of the potato bivalent mark-associated genes. Figure S14. Bivalent histone modification levels and expression levels of the potato bivalent mark-associated genes in cold tubers. Figure S15. DNase I sensitivity of the bivalent mark-associated genes in potato tubers upon cold stress. Figure S16. Expression levels of putative PcG and TrxG genes in potato tubers upon cold stress. Figure S17. Histone modification H3K4me3 associated with the bivalent mark-associated genes in potato tubers upon cold stress. (PDF 1782 kb) [file 13059_2019_1731_MOESM1_ESM.pdf]

Additional file 1: Figures S1-S17

Cold stress induces enhanced chromatin accessibility and bivalent histone modifications H3K4me3 and H3K27me3 of active genes in potato

Zixian Zeng[1,2,3,4], Wenli Zhang[1,5], Alexandre P. Marand[1], Bo Zhu[4], C. Robin Buell[2], Jiming Jiang[1,2,3,6*]

[1] Department of Horticulture, University of Wisconsin-Madison, Madison, Wisconsin 53706, USA

[2] Department of Plant Biology, Michigan State University, East Lansing, Michigan 48824, USA

[3] Department of Horticulture, Michigan State University, East Lansing, Michigan 48824, USA

[4] Department of Biological Science, College of Life Sciences, Sichuan Normal University, Chengdu, Sichuan 610101, China

[5] State Key Laboratory for Crop Genetics and Germplasm Enhancement, Nanjing Agriculture University, Nanjing, Jiangsu 210095, China

[6] Michigan State University AgBioResearch, East Lansing, MI 48824, USA

*Address correspondence to: jiangjm@msu.edu

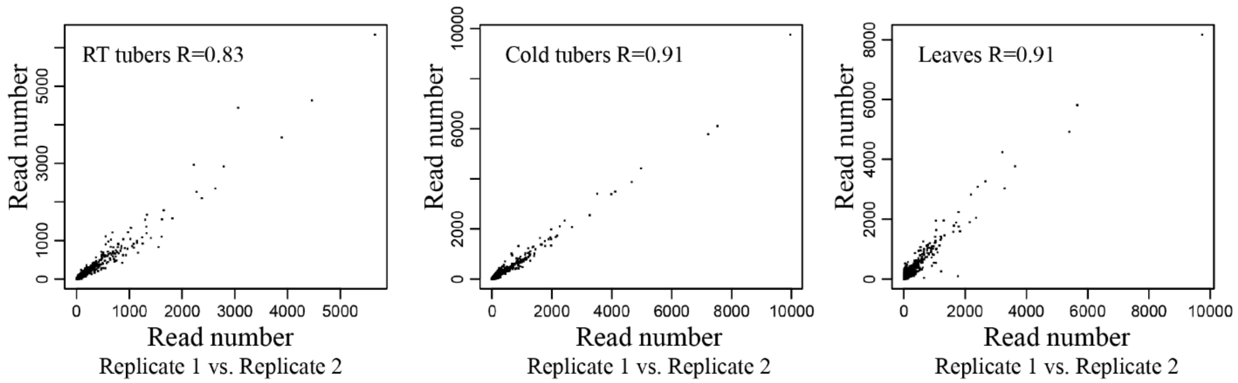

Figure S1. DNase-seq data correlation between two biological replicates derived from RT tubers, cold tubers and leaves. The entire potato genome was divided into 100 bp non-overlapping windows. The number of DNase-seq reads per window normalized to the total mapped reads was used to examine the correlation in sequence coverage between replicates. Pearson correlation coefficients are shown in the upper left window for each library.

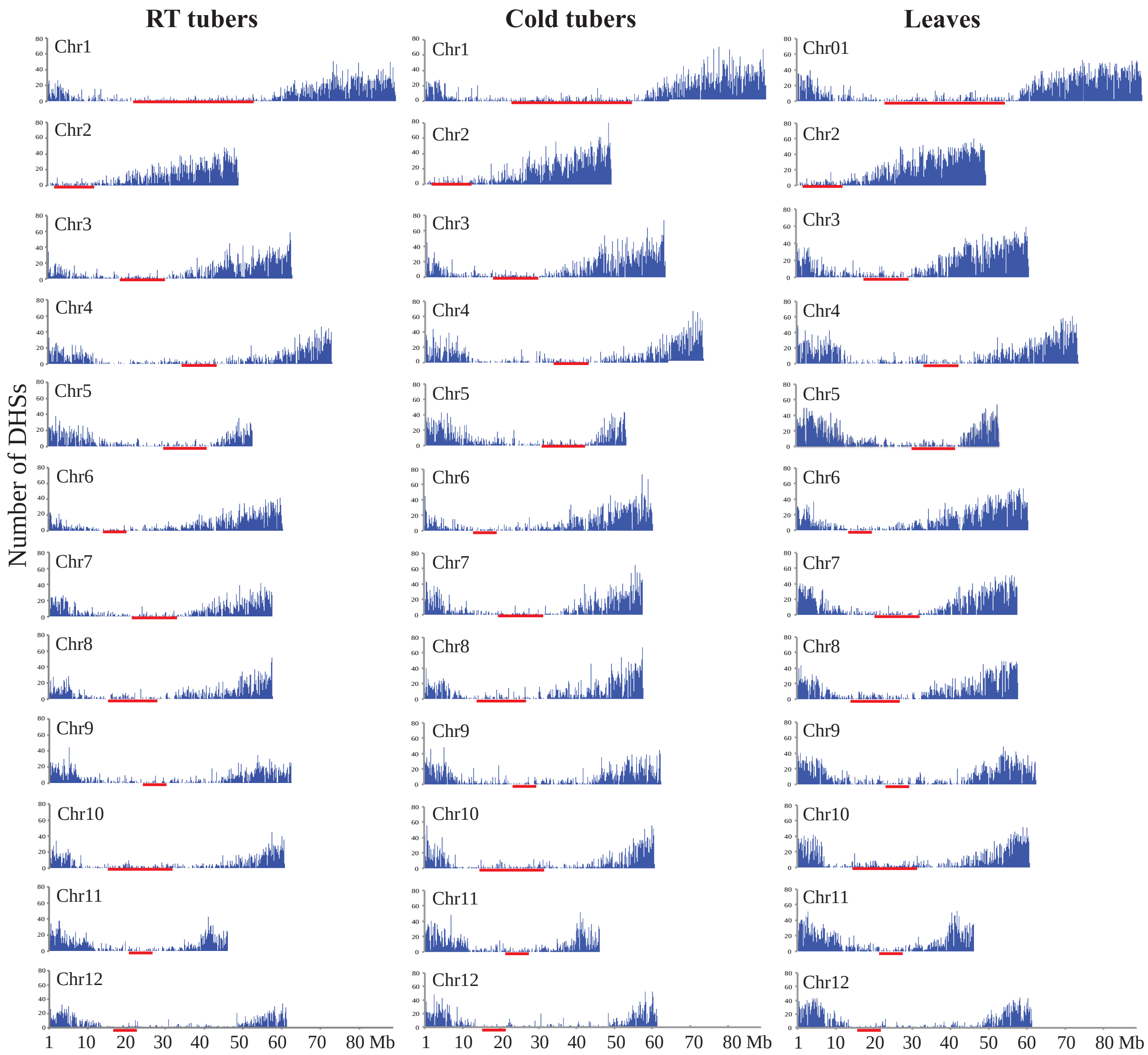

Figure S2. Distribution of DHSs on all potato chromosomes. The *x*-axis indicates the physical position on the chromosome in megabases (Mb). The *y*-axis indicates the number of DHSs per 10 kb window. The red horizontal bars indicate putative centromeric regions [1].

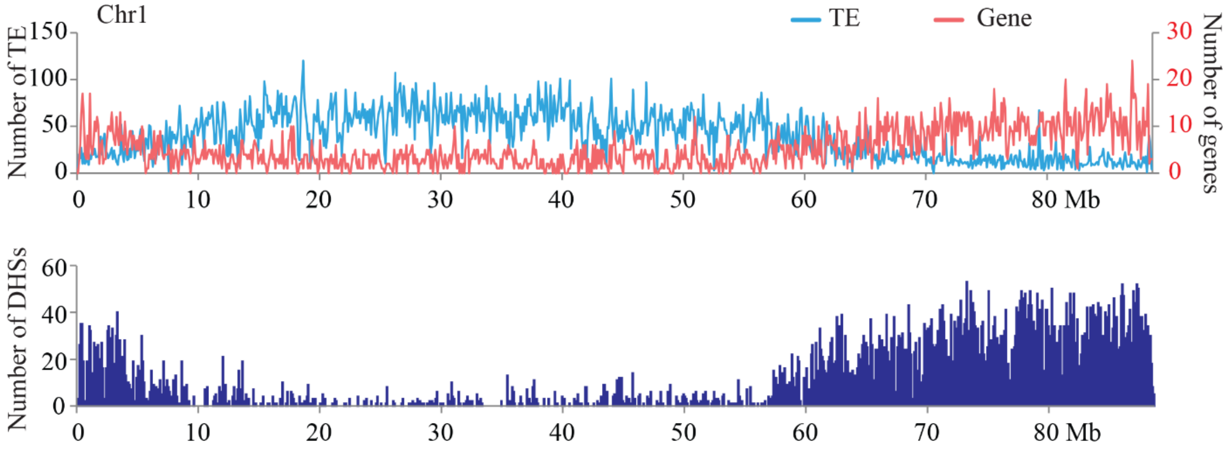

Figure S3. Distribution of DHSs derived from potato leaves. Top panel: distribution of transposable elements (TEs) and genes along potato chromosome 1. The left *y*-axis indicates the number of TEs (blue line) and the right *y*-axis indicates the number of genes (red line). Bottom panel: distribution of DHSs along potato chromosome 1. The *y*-axis indicates the number of the DHSs. The number of DHSs, TE and genes was calculated for each non-overlapping 10 kb window across chromosome 1. The *x*-axis indicates the physical position of chromosome 1 in megabases.

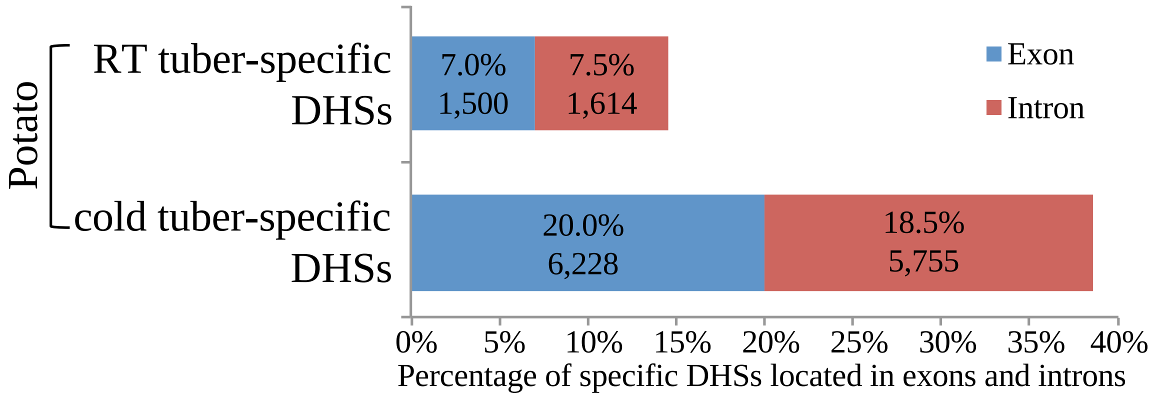

Figure S4. Comparison in number of temperature-specific genic DHSs between RT and cold tubers. The percentage and the number of total RT tuber- and cold tuber-specific DHSs located in exons and introns, respectively.

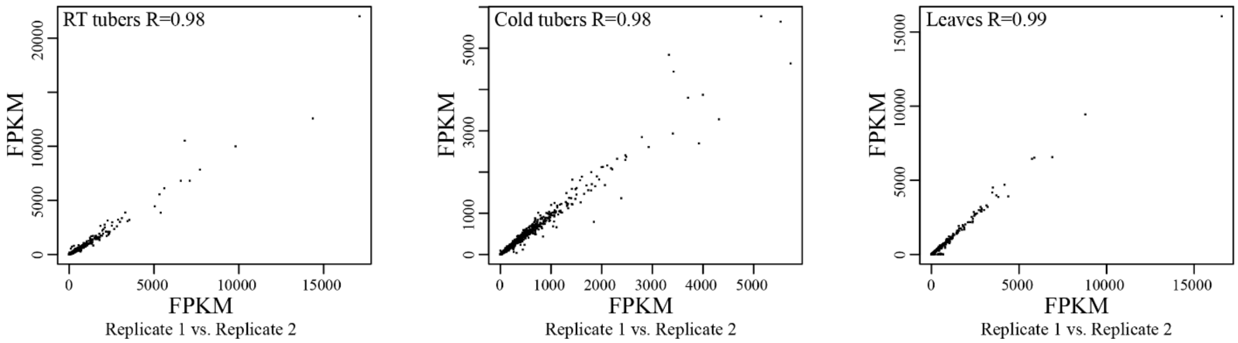

Figure S5. RNA-seq data correlations between two biological replicates derived from RT tubers, cold tubers and leaves. FPKM values for 39,400 annotated potato genes were used to evaluate the correlation between replicates. Pearson correlation coefficients are show in the upper left window for each library.

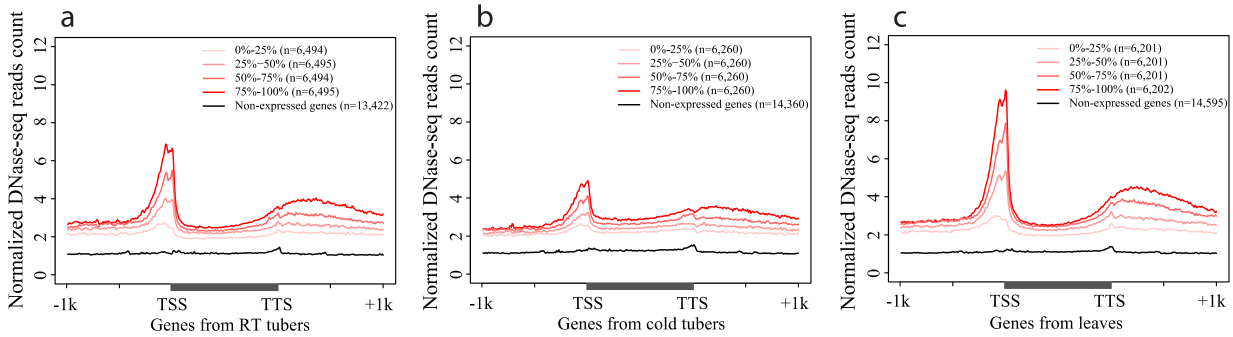

Figure S6. Relationship between DNase I sensitivity and gene expression levels in potato. The profile of DNase I sensitivity among genes with different expression levels in RT tubers **a**, cold tubers **b**, and leaves **c**. Active genes were divided into 4 groups and sorted from low expression (0%-25%) to high expression (75%-100%) according to their expression levels (FPKM) in each sample. Non-expressed genes were genes with FPKM equal to 0 in each sample. Genes were divided into 100 bins and aligned together from TSSs to TTSs. Genes flanking regions (±1 kb) were analyzed in 100 bins. The *y*-axis shows the normalized average number of DNase-seq reads per bp genome region per billion reads.

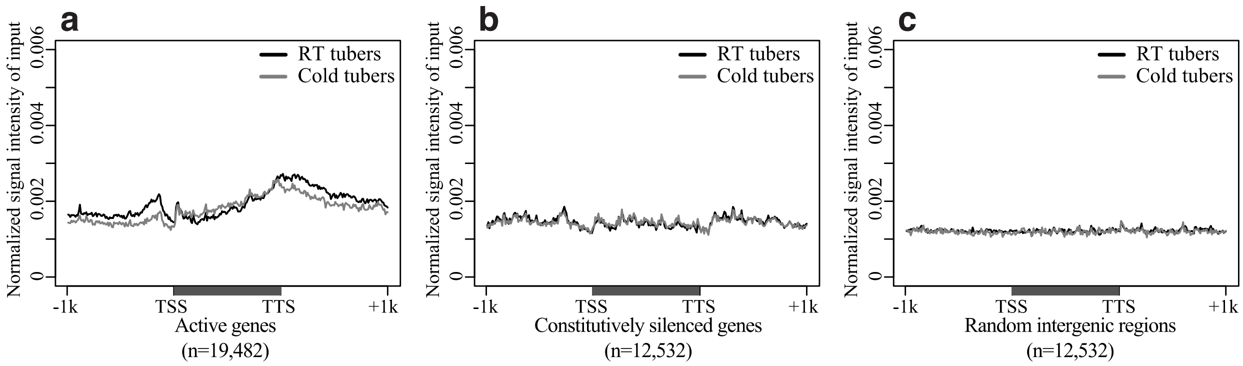

Figure S7. The nucleosome density associated with potato genes. Similar input signal intensity for active genes **a** (Pearson correlation r=0.84), constitutively silenced genes **b** (Pearson correlation r=0.85) and random intergenic regions **c** (Pearson correlation r=0.47) between RT and cold tubers. Each active gene showed transcription (FPKM >1) in both RT and cold tubers. Constitutively silenced genes did not show transcription in either RT or cold tubers (FPKM=0). "Random intergenic regions" were randomly selected from regions that were at least 2 kb away from any annotated genes. The length and number of the random intergenic regions were the same as constitutively silenced genes. Nucleosome density is measured by input read number per bp genome region per million mapped reads. Genes were divided into 100 bins and aligned together from TSSs to TTSs. Genes flanking regions (±1 kb) were analyzed in 100 bins.

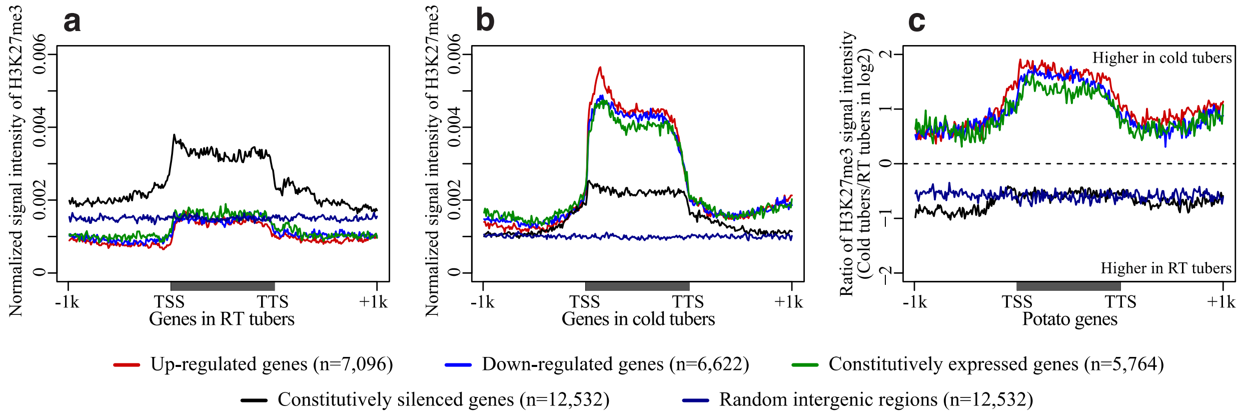

Figure S8. Profiles of histone modifications associated with potato genes upon cold stress. Profiles of H3K27me3 for the potato genes in RT tubers **a** and cold tubers **b**. **c** The ratio of H3K27me3 signal intensity between cold vs. RT tubers. The same set of up-regulated (n=7,096), down-regulated (n=6,622), constitutively expressed genes (n=5,764) and constitutively silenced genes (n=12,532) were used in all analyses. Constitutively silenced genes did not show transcription in either RT or cold tubers (FPKM=0). “Random intergenic regions” were randomly selected from regions that were at least 2 kb away from any annotated genes. The length and number of the random intergenic regions were the same as constitutively silenced genes. The histone modification signal was normalized by ChIP-seq read number per bp genome region per million mapped reads. Genes were divided into 100 bins and aligned together from TSSs to TTSs. Genes flanking regions (±1 kb) were analyzed in 100 bins.

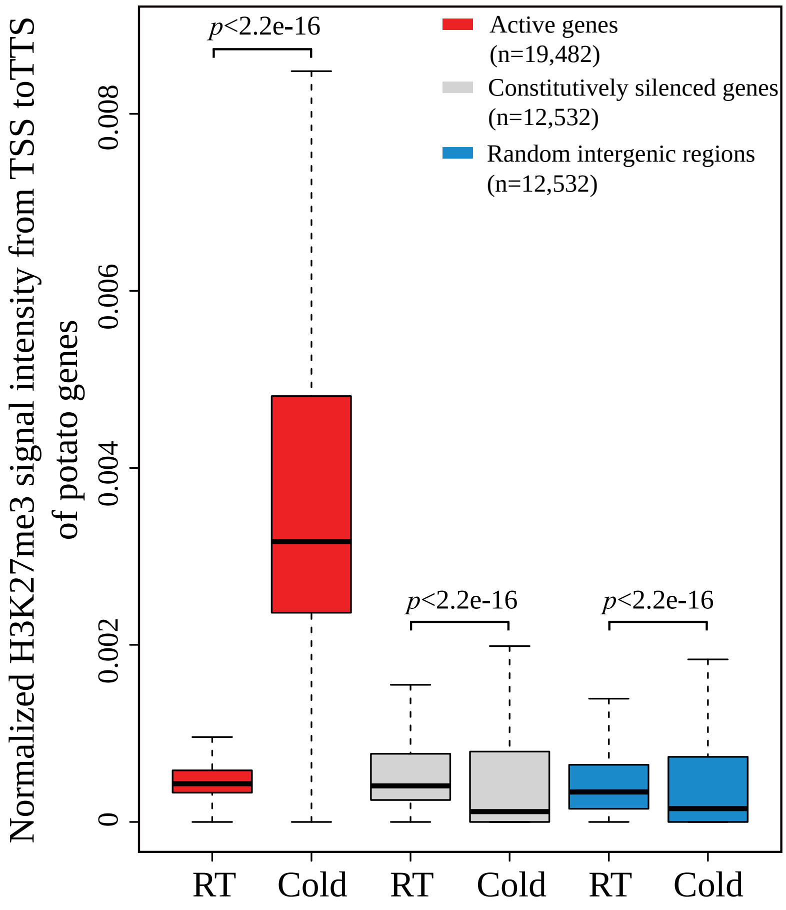

Figure S9. H3K27me3 levels of potato genes between RT and cold tubers. Signal intensity of H3K27me3 was measured from transcription start site (TSS) to transcription terminate site (TTS) for each gene. Each active gene showed transcription (FPKM>1) in both RT and cold tubers. Constitutively silenced genes did not display transcription in either RT or cold tubers (FPKM=0). "Random intergenic regions" were randomly selected from regions that were at least 2 kb away from any annotated genes. The length and number of the random intergenic regions were the same as constitutively silenced genes. RT indicates RT tubers and Cold indicates cold tubers. Statistical significance was tested using Wilcoxon Rank Sum test.

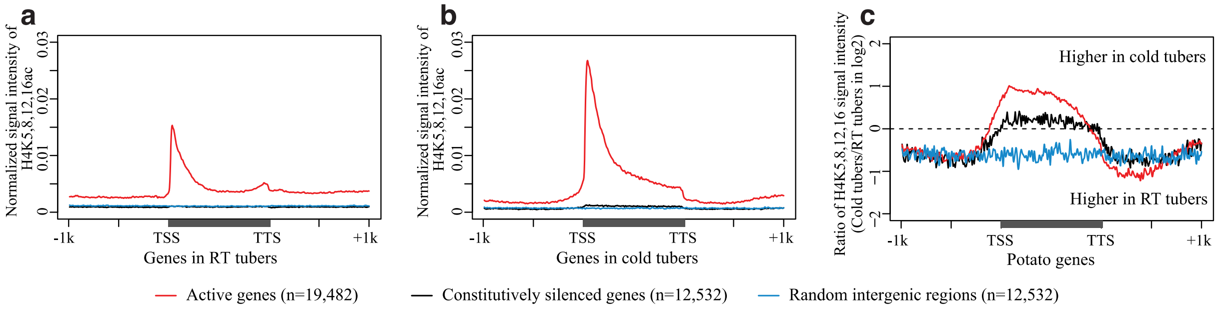

Figure S10. Profiles of histone modification H4K5, 8, 12, 16ac associated with potato genes. Profile of H4K5,8,12,16ac for active genes in RT tubers **a** and cold tubers **b**. **c** The ratio of H4K5,8,12,16ac signal intensity between cold vs. RT tubers. The same set of active genes (n=19,482) and constitutively silenced genes (n=12,532) were used in all analyses. Each active gene showed transcription (FPKM >1) in both RT and cold tubers. Constitutively silenced genes did not show transcription in either RT or cold tubers (FPKM=0). “Random intergenic regions” were randomly selected from regions that were at least 2 kb away from any annotated genes. The length and number of the random intergenic regions were the same as constitutively silenced genes. Genes were divided into 100 bins and aligned together from TSSs to TTSs. Genes flanking regions (±1 kb) were analyzed in 100 bins. The histone modification signal was normalized by ChIP-seq read number per bp genome region per million mapped reads.

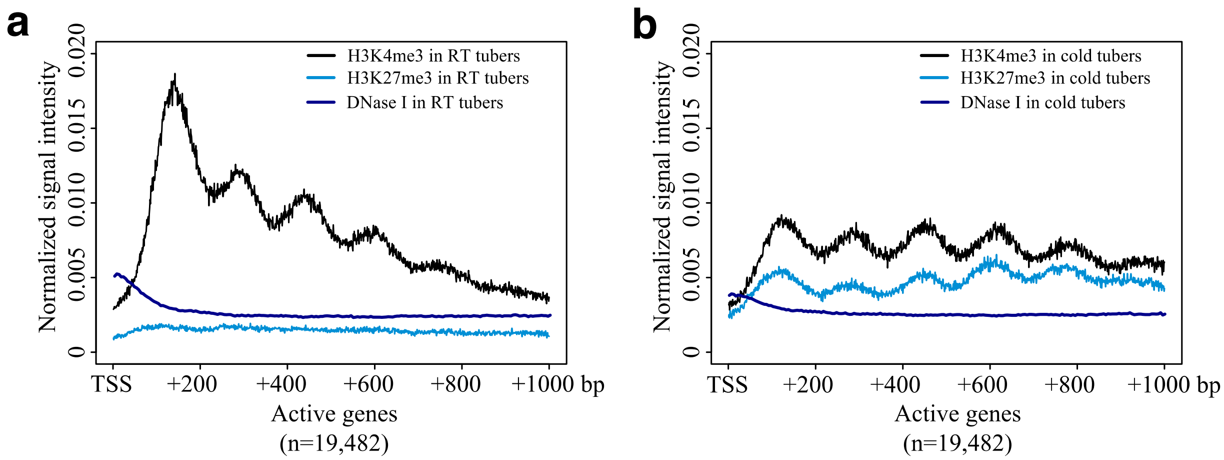

Figure S11. **Histone modifications associated with active genes in cold-stressed tubers.** Profiles of H3K4me3, H3K27me3 and DNase I signal intensity across active genes in RT tubers **a** and cold tubers **b**. Each active gene showed transcription (FPKM >1) in both RT and cold tubers. Only the first 1000 bp sequence (from TSS) of each gene was used to calculate histone modifications and DNase I signal intensity.

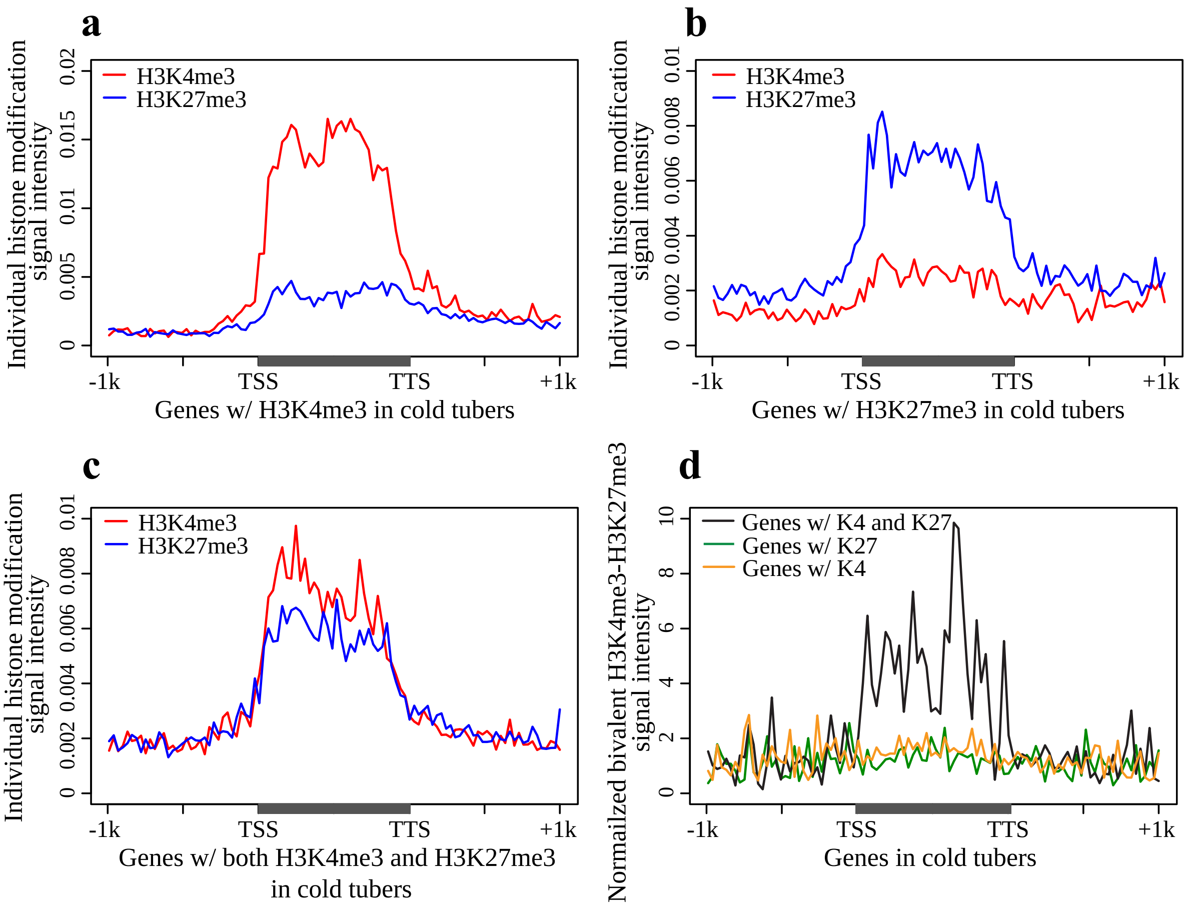

Figure S12. Profiles of histone modifications associated with potato genes in cold tubers. Individual histone modification levels for genes that mainly associated with H3K4me3 **a**, H3K27me3 **b,** and both H3K4me3 and H3K27me3 **c**. **d** Bivalent H3K4me3-H3K27me3 histone modification levels for three groups of genes that associated with H3K4me3, H3K27me3 as well as both individual H3K4me3 and H3K27me3 marks, respectively. The same number of genes (n=400) were selected for each gene group. Each gene showed transcription (FPKM >1) in cold tubers. Genes were divided into 100 bins and aligned together from TSSs to TTSs. Genes flanking regions (±1 kb) were analyzed in 100 bins. Histone modification signal was normalized by ChIP-seq read number per bp genome region per million mapped reads. Sequential K4-K27 ChIP-seq signal was normalized by control data K4-noAb and by sequential ChIP-seq read number per bp genome region per million mapped reads.

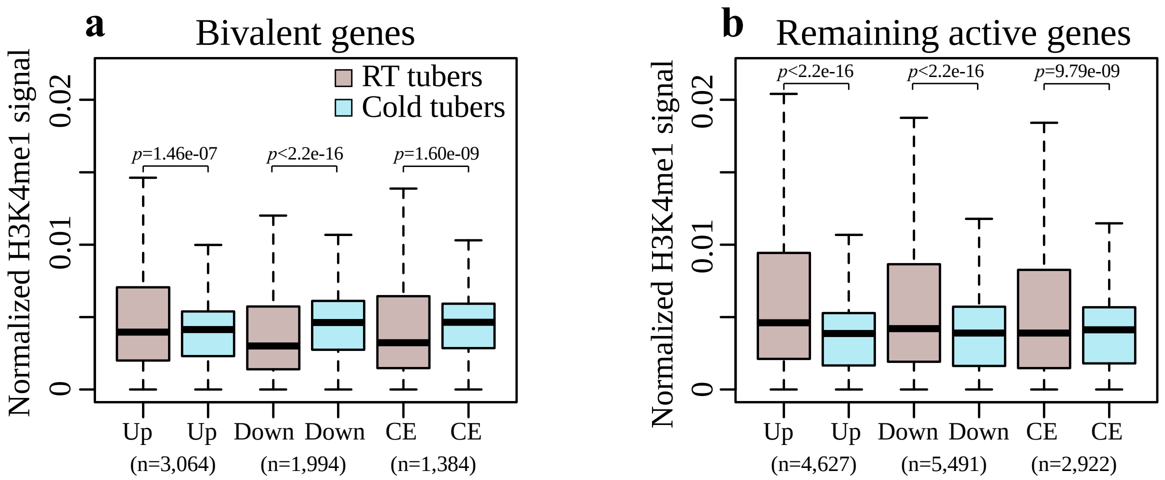

Figure S13. Histone modification H3K4me1 levels of the potato bivalent marks-associated genes. H3K4me1 levels in gene body regions of the potato bivalent marks-associated genes (6,442) **a** and the remaining active genes (13,040) **b** between RT and cold tubers. Signal intensity of H3K4me1 was measured from transcription start site (TSS) to transcription terminate site (TTS) for each gene and normalized by ChIP-seq read number per bp genome region per million mapped reads. Up, down and CE indicates up-regulated, down-regulated and constitutively expressed genes, respectively, upon cold stress. Statistical significance was tested using Wilcoxon Rank Sum test.

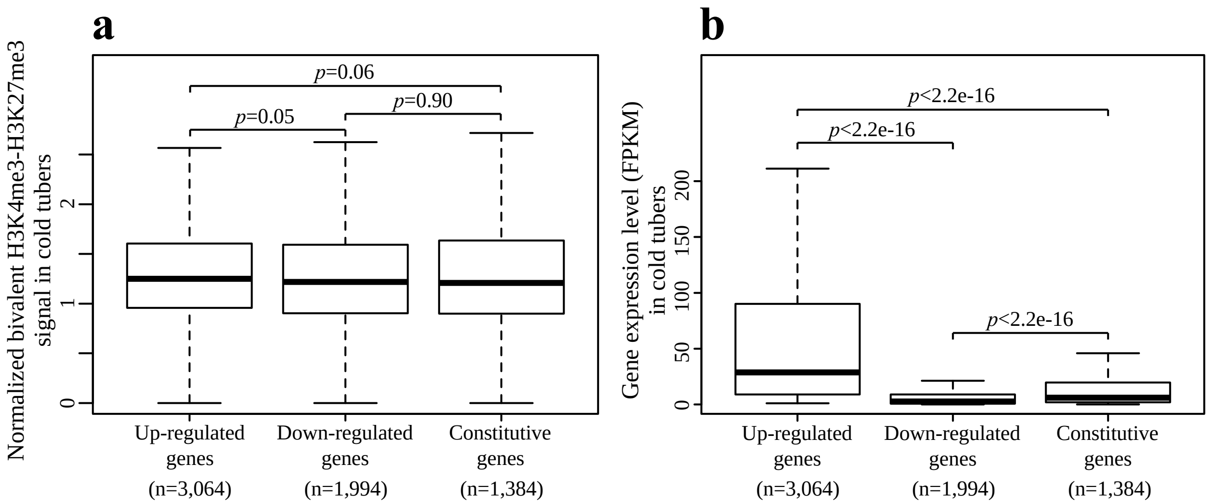

Figure S14. Bivalent histone modification levels and expression levels of the potato bivalent marks-associated genes in cold tubers. a Bivalent H3K4me3-H3K27me3 modification levels in gene body regions of the potato bivalent marks-associated genes (6,442) in cold tubers. **b** Gene expression levels of the potato bivalent marks-associated genes in cold tubers. Bivalent modifications signal intensity was measured using sequential K4-K27 ChIP-seq data from transcription start site (TSS) to transcription terminate site (TTS) for each gene and normalized by the control sequential ChIP-seq data K4-noAb, and by sequential ChIP-seq read number per bp genome region per million mapped reads. Bivalent marks-associated genes were categorized into three groups according to their expression upon cold stress. Including up-regulated, down-regulated and constitutively expressed genes. Statistical significance was tested using Wilcoxon Rank Sum test.

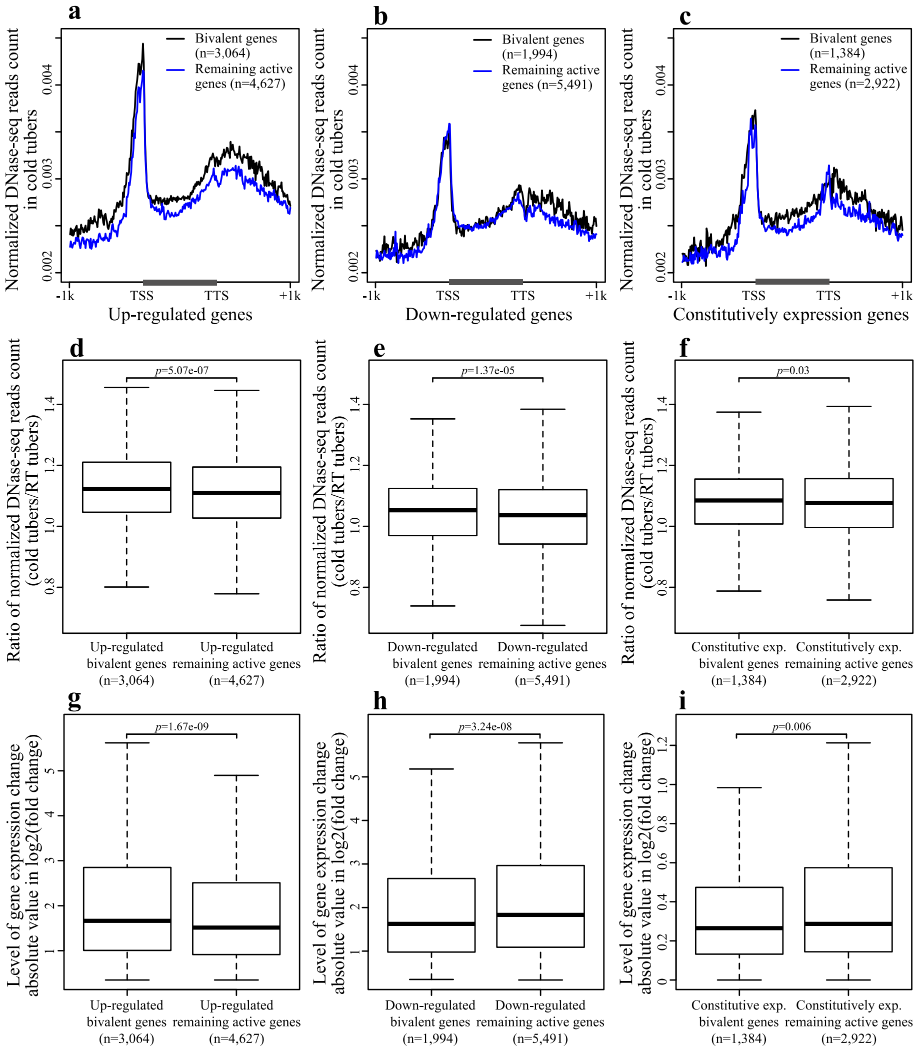

Figure S15. DNase I sensitivity of the bivalent marks-associated genes in potato tubers upon cold stress. Comparison of DNase I sensitivity between the up-regulated bivalent marks-associated genes and the remaining up-regulated genes **a**, between down-regulated bivalent marks-associated genes and the remaining down-regulated genes **b,** and between constitutively expressed bivalent marks-associated genes and the remaining constitutively expressed genes **c** in cold tubers. Genes were divided into 100 bins and aligned together from TSSs to TTSs. Genes flanking regions (±1 kb) were analyzed in 100 bins. The levels of DNase I sensitivity elevation between up-regulated bivalent marks-associated genes and the remaining up-regulated genes **d**, between down-regulated bivalent marks-associated genes and the remaining down-regulated genes **e**, and between constitutively expressed bivalent marks-associated genes and the remaining constitutively expressed genes **f** upon cold stress. DNase I sensitivity was measured from transcription start site (TSS) to transcription terminate site (TTS) for each gene. DNase I sensitivity was normalized by the number of DNase-seq reads per bp genome region per billion reads. Fold changes of gene expression in log2 upon cold stress between up-regulated bivalent marks-associated genes and the remaining up-regulated genes **g**, between down-regulated bivalent marks-associated genes and the remaining down-regulated genes **h**, and between constitutively expressed bivalent marks-associated genes and the remaining constitutively expressed genes **i.** Statistical significance was tested using Wilcoxon Rank Sum test.

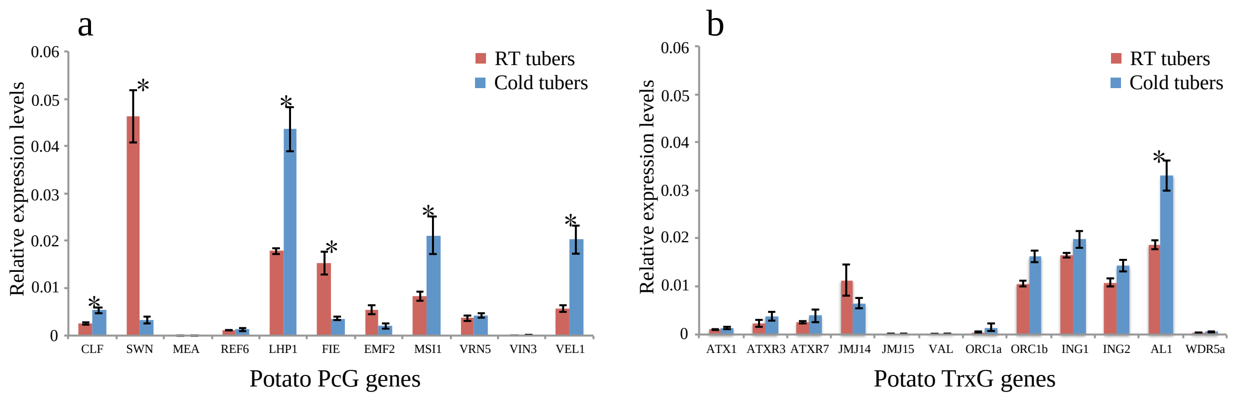

Figure S16. Expression levels of putative PcG and TrxG genes in potato tubers upon cold stress. a Comparison of PcG genes expression between RT and cold tubers. **b** Comparison of TrxG genes expression between RT and cold tubers. Expression levels of PcG and TrxG genes were quantified and estimated relative to reference gene *EF1α*. Error bar indicates standard deviation. *t-test p<0.01.

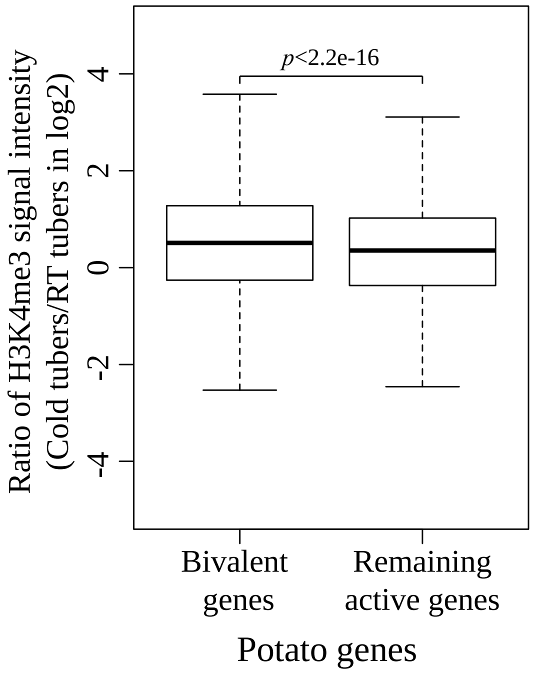

Figure S17. Histone modification H3K4me3 associated with the bivalent marks-associated genes in potato tubers upon cold stress. The ratio of H3K4me3 signal intensity for the bivalent marks-associated genes (6,442) between cold tubers vs. RT tubers as well as for the remaining active genes (13,040). H3K4me3 signal intensity was measured from transcription start site (TSS) to transcription terminate site (TTS) for each gene and was normalized by the number of DNase-seq reads per bp genome region per billion reads. Statistical significance was tested using Wilcoxon Rank Sum test.

Reference

1. Gong Z, Wu Y, Koblizkova A, Torres GA, Wang K, Iovene M, Neumann P, Zhang W, Nova k P, Buell CR, et al: **Repeatless and repeat-based centromeres in potato: implications for centromere evolution.** *Plant Cell* 2012, **24**:3559-3574.
