# Supplementary material for: Cold stress induces enhanced chromatin accessibility and bivalent histone modifications H3K4me3 and H3K27me3 of active genes in potato
Source: Genome Biol. 2019 Jun 17;20:123. doi: 10.1186/s13059-019-1731-2 (PMC6580510; doi:10.1186/s13059-019-1731-2)
Supplement: Supplementary file 2 — Table S1. DNase-seq data generated from potato RT tubers, cold tubers, and leaves. Table S2. RNA-seq data generated from potato RT tubers, cold tubers, and leaves. Table S3. Differentially expressed genes involved in potato tuber carbohydrate pathway under cold stress. Table S4. Differentially expressed genes involved photosynthesis in potato. Table S5. ChIP-seq and sequential ChIP-seq data generated from potato DM. Table S6. Expression of the genes associated with trimethylation of H3K27 in RT and 14-day cold-treated potato tubers. Table S7. Expression of the genes associated with trimethylation of H3K4 in RT and 14-day cold-treated potato tubers. Table S8. Primers used for qRT-PCR. (PDF 201 kb) [file 13059_2019_1731_MOESM2_ESM.pdf]

Additional file 2: Tables S1-S8

Cold stress induces enhanced chromatin accessibility and bivalent histone modifications H3K4me3 and H3K27me3 of active genes in potato

Zixian Zeng[1,2,3,4], Wenli Zhang[1,5], Alexandre P. Marand[1], Bo Zhu[4], C. Robin Buell[2], Jiming Jiang[1,2,3,6*]

[1] Department of Horticulture, University of Wisconsin-Madison, Madison, Wisconsin 53706, USA

[2] Department of Plant Biology, Michigan State University, East Lansing, Michigan 48824, USA

[3] Department of Horticulture, Michigan State University, East Lansing, Michigan 48824, USA

[4] Department of Biological Science, College of Life Sciences, Sichuan Normal University, Chengdu, Sichuan 610101, China

[5] State Key Laboratory for Crop Genetics and Germplasm Enhancement, Nanjing Agriculture University, Nanjing, Jiangsu 210095, China

[6] Michigan State University AgBioResearch, East Lansing, MI 48824, USA

*Address correspondence to: jiangjm@msu.edu

Table S1. DNase-seq data generated from potato RT tubers, cold tubers and leaves.

SRA accession number	Tissue	Treatment	Replicate	Total # reads	# reads mapped	% reads mapped	# reads uniquely mapped	% reads uniquely mapped
SRR5242711	Tuber	4ºC for 14 days	Replicate1	84,903,663	78,988,592	93.0%	38,015,402	44.77%
SRR5242710	Tuber	4ºC for 14 days	Replicate2	98,815,306	93,232,154	94.4%	46,837,393	47.40%
SRR5242709	Tuber	RT for 14 days	Replicate1	78,497,400	71,046,863	89.4%	32,355,147	40.71%
SRR5242708	Tuber	RT for 14 days	Replicate2	95,900,574	90,307,264	94.4%	47,379,610	49.53%
SRR5242707	Leaf	None	Replicate1	86,593,534	83,570,011	96.7%	44,226,054	51.19%
SRR5242706	Leaf	None	Replicate2	78,351,335	75,780,242	96.7%	39,910,091	50.94%

Table S2. RNA-seq data generated from potato RT tubers, cold tubers and leaves.

SRA accession number	Tissue	Treatment	Replicate	Total # reads	# reads uniquely mapped	% reads uniquely mapped
SRR5242705	Tuber	4ºC for 14 days	Replicate1	51,099,106	44,320,641	86.7%
SRR5242704	Tuber	4ºC for 14 days	Replicate2	62,088,596	55,664,557	89.7%
SRR5242703	Tuber	RT for 14 days	Replicate1	77,937,797	65,066,599	83.5%
SRR5242702	Tuber	RT for 14 days	Replicate2	70,882,989	55,663,405	78.5%
SRR5242701	Leaf	None	Replicate1	19,364,935	13,946,760	71.5%
SRR5242700	Leaf	None	Replicate2	38,081,192	26,406,707	69.3%

Table S3. Differentially expressed genes involved in potato tuber carbohydrate pathway under cold stress.

Locus	Gene_name	Log2(fold change) (cold tubers/RT tubers)	Expresssion value in RT tubers	Expresssion value in cold tubers
PGSC0003DMG400033858	Alpha-glucan phosphorylase, L-type, plastidic, Stp23	-6.07712	1000.52	14.8193
PGSC0003DMG400020699	Debranching enzyme, isoamylase isoform 1	-5.90193	52.6382	0.880322
PGSC0003DMG400005269	Glucose-6-phosphate/phosphate translocator, plastid	-5.66095	556.744	11.0037
PGSC0003DMG400013547	Sucrose synthase	-5.34164	1.59192	0.0392579
PGSC0003DMG400003495	Alpha-glucan phosphorylase, L-type, plastidic, Stp23	-5.32689	2073.05	51.6482
PGSC0003DMG400007782	Alpha-glucan phosphorylase, L-type, plastidic, Stp23	-5.32689	1459.74	33.1114
PGSC0003DMG400031084	ADP-glucose pyrophosphorylase AGP	-5.29461	366.434	9.33599
PGSC0003DMG400002479	Alpha-glucan phosphorylase, L-type, plastidic, Stp23	-5.27636	1900.05	49.0253
PGSC0003DMG400009981	Starch branching enzyme I	-4.62599	364.106	14.7457
PGSC0003DMG400030253	Debranching enzyme, isoamylase isoform 1	-4.60659	16.965	0.696356
PGSC0003DMG400000735	ADP-glucose pyrophosphorylase AGP	-4.47375	496.966	22.3663
PGSC0003DMG400009891	Alpha-amylase, AmyZ	-4.10777	348.456	20.2109
PGSC0003DMG400000954	Debranching enzyme, isoamylase isoform 2	-3.87385	27.9951	1.90958
PGSC0003DMG400012111	Granule bound starch synthase I, plastidic, waxy	-3.67775	596.251	46.5924
PGSC0003DMG400024812	Maltose transporter	-3.66565	42.8329	3.37526
PGSC0003DMG400031046	Sucrose synthase	-3.55355	12.8297	1.09268
PGSC0003DMG400016481	Starch synthase, soluble, plastidic	-3.51789	21.8683	1.90909
PGSC0003DMG400030619	Starch synthase V	-3.28442	23.1694	2.37797
PGSC0003DMG400013187	Hexokinase	-2.96191	4.17336	0.535629
PGSC0003DMG400016589	Disproportionating enzyme, 4-alpha glucanotransferase plastidic	-2.83332	138.491	19.4316
PGSC0003DMG400015246	Phosphoglucan phosphatase	-2.74998	159.404	23.6958
PGSC0003DMG400001328	Granule bound starch synthase II, plastidic, SSII	-2.67247	39.3666	6.17495
PGSC0003DMG400007677	Alpha-glucan water dikinase	-2.65162	256.538	40.8258
PGSC0003DMG401007274	Debranching enzyme, isoamylase isoform 3	-2.59678	76.6436	12.6697
PGSC0003DMG400015952	ADP-glucose pyrophosphorylase AGP	-2.36082	22.0699	4.29657
PGSC0003DMG400002895	Sucrose synthase, Sus4	-2.32583	815.111	162.582
PGSC0003DMG400028311	Fructokinase	-2.32471	4.41661	0.881622
PGSC0003DMG400030624	Hexokinase	-2.23638	10.4022	2.20753
PGSC0003DMG400026530	Invertase, beta-fructofuranosidase, neutral	-2.12099	26.422	6.0741
PGSC0003DMG400009994	Hexose transporter	-2.10079	19.9364	4.64778
PGSC0003DMG400016613	Phosphoglucan water dikinase	-2.01722	91.1253	22.511
PGSC0003DMG401013333	UDP-glucose pyrophosphorylase	-1.9627	1007.85	258.561
PGSC0003DMG402007274	Debranching enzyme, isoamylase isoform 3	-1.92813	91.3319	23.9992
PGSC0003DMG401013540	Starch synthase VI	-1.91415	10.265	2.7236
PGSC0003DMG400004616	Invertase inhibitor	-1.79835	229.459	65.97
PGSC0003DMG402013540	Starch synthase VI	-1.64198	11.5563	3.70283
PGSC0003DMG400026428	Sucrose phosphate synthase isoform B	-1.59637	4.03878	1.33566

PGSC0003DMG400000169	Beta-amylase	-1.46403	7.03232	2.54908
PGSC0003DMG400001844	Invertase inhibitor	-1.08605	1.30928	0.616735
PGSC0003DMG400005602	Glucose-6-phosphate/phosphate translocator, plastidic	-0.960277	222.967	114.596
PGSC0003DMG400001041	Glucose-6-phosphate/phosphate translocator, plastid	-0.851535	246.023	136.345
PGSC0003DMG400009257	Invertase, beta-fructofuranosidase, neutral /alkaline	-0.724309	5.92351	3.58542
PGSC0003DMG400015341	Glucose-6-phosphate isomerase	-0.687458	111.413	69.1818
PGSC0003DMG400004617	Invertase inhibitor	-0.525726	133.649	92.833
PGSC0003DMG400027017	Fructokinase	-0.512873	78.4199	54.9586
PGSC0003DMG400026916	Fructokinase, StFrk2	-0.382701	290.58	222.875
PGSC0003DMG400009213	Sucrose transporter	0.405517	42.2741	55.9948
PGSC0003DMG400001912	Phosphoglucomutase, plastidic	0.58863	20.77	31.2343
PGSC0003DMG400027936	Sucrose phosphate synthase	0.5953	133.573	201.801
PGSC0003DMG401017626	Alpha-amylase	1.01599	16.345	33.0543
PGSC0003DMG400022402	Hexose transporter	1.08027	4.86627	10.2894
PGSC0003DMG401031123	UDP-glucose pyrophosphorylase	1.0911	14.1759	30.1997
PGSC0003DMG400026107	Invertase, beta-fructofuranosidase, neutral /alkaline	1.10789	13.8218	29.79
PGSC0003DMG400024224	Phosphoglucomutase, plastidic	1.35639	8.4905	21.7394
PGSC0003DMG400013088	Invertase, beta-fructofuranosidase, neutral	1.49477	27.0798	76.316
PGSC0003DMG400010664	Beta-amylase	1.49963	170.483	482.077
PGSC0003DMG400002525	Hexokinase	1.57681	20.089	59.9275
PGSC0003DMG402012710	Glucose-6-phosphate/phosphate translocator, plastid	1.69994	1.89453	6.15508
PGSC0003DMG400033142	Invertase, beta-fructofuranosidase, cell wall	1.857	0.433364	1.56989
PGSC0003DMG400012910	Glucose-6-phosphate isomerase	2.02153	12.394	50.3213
PGSC0003DMG400019494	Invertase, beta-fructofuranosidase	2.14356	10.9487	48.376
PGSC0003DMG400024246	Fructokinase, StFrk1	2.15064	40.2909	178.902
PGSC0003DMG402018758	Hexose transporter	2.16007	3.08912	13.8065
PGSC0003DMG400009936	Invertase, beta-fructofuranosidase	2.26519	17.6408	84.802
PGSC0003DMG400025610	Sucrose transporter	2.47112	6.25187	34.665
PGSC0003DMG400002756	Invertase, beta-fructofuranosidase	2.48614	6.08079	34.069
PGSC0003DMG402013388	Amylase inhibitor	2.67794	11.6256	74.3972
PGSC0003DMG400001855	Beta-amylase, platidic PCT-BMYI	3.33633	4.85239	49.0108
PGSC0003DMG400031832	Hexose transporter	3.67081	5.34123	68.0245
PGSC0003DMG400013546	Sucrose synthase, Sus3	4.02782	69.3899	1131.85
PGSC0003DMG400016730	Sucrose synthase	4.92865	0.192812	5.87223
PGSC0003DMG400004790	Invertase, beta-fructofuranosidase	7.04391	0.687061	90.6618
PGSC0003DMG402028252	Invertase, beta-fructofuranosidase, cell wall	7.44894	0.691433	120.811

Table S4. Differentially expressed genes involved photosynthesis in potato.

Locus	Gene_name	Log2(fold change) (RT tubers/leaves)	Expresssion value in leaves	Expresssion value in RT tubers
PGSC0003DMG400011530	Glyceraldehyde-3-phosphate dehydrogenase A, chloroplastic	-14.8109	1661.69	0.698507
PGSC0003DMG400019584	Ribulose bisphosphate carboxylase small chain 1, chloroplastic	-14.5311	2893.43	7.33452
PGSC0003DMG400019508	Chorophyll a/b binding protein	-13.6757	1847.9	1.61044
PGSC0003DMG400013460	Chlorophyll a-b binding protein 3C, chloroplastic	-13.4267	23.0106	0.718178
PGSC0003DMG400017556	Photosystem II 22 kDa protein, chloroplastic	-12.8508	4898.54	32.8189
PGSC0003DMG400023344	Chlorophyll a-b binding protein 6A, chloroplastic	-12.7762	3068.41	2.62383
PGSC0003DMG400009042	Chloroplasti CP12	-12.7385	18994.2	0.802261
PGSC0003DMG400027672	Photosystem I subunit XI	-12.3807	3405.86	15.4612
PGSC0003DMG400027671	Photosystem I subunit XI	-12.223	3701.18	0.28285
PGSC0003DMG400019248	Chlorophyll a-b binding protein 13, chloroplastic	-12.141	666.744	0.298467
PGSC0003DMG400005890	16kDa membrane protein	-11.9604	29.8814	1.06824
PGSC0003DMG400026500	Type I (26 kD) CP29 polypeptide	-11.6818	2126.38	2.72792
PGSC0003DMG400019149	Ribulose bisphosphate carboxylase/oxygenase activase, chloroplastic	-11.3967	2759.25	4.91835
PGSC0003DMG400007201	Photosystem II core complex proteins psbY, chloroplast	-11.2161	50.4282	2.28959
PGSC0003DMG400020505	Photosystem I reaction center subunit X psaK	-11.1253	1745.77	0.158544
PGSC0003DMG400002782	Oxygen-evolving enhancer protein 1, chloroplastic	-11.0687	2751.19	1.28087
PGSC0003DMG400016504	PSI-H	-10.8521	2150.9	1.79874
PGSC0003DMG400021727	Photosystem II oxygen-evolving complex protein 3	-10.3734	38.769	2.58003
PGSC0003DMG400008585	Photosystem II reaction center psb28 protein	-10.327	7852.96	90.6105
PGSC0003DMG400006149	Chlorophyll a-b binding protein 4, chloroplastic	-10.2237	511.853	0.398476
PGSC0003DMG400014386	Chlorophyll a/b binding protein 7, chloroplastic	-10.1916	4.66566	0.770169
PGSC0003DMG400005805	Photosystem I reaction center subunit	-10.1642	520.326	10.3114
PGSC0003DMG400010035	Oxygen-evolving enhancer protein 1, chloroplastic	-9.60638	2276.69	0.571306
PGSC0003DMG400020141	Photosystem II reaction center W protein, chloroplastic	-9.46311	1812.12	3.04713
PGSC0003DMG402003567	Ferredoxin--NADP reductase, leaf-type isozyme, chloroplastic	-9.21601	2151.84	0
PGSC0003DMG400021287	Chlorophyll a/b binding protein 8, chloroplastic	-9.13188	1746.21	7.60645
PGSC0003DMG400022249	Chloroplast photosystem I reaction center V	-9.11981	3352.17	1.24331
PGSC0003DMG400011816	Photosystem I reaction centre PSI-D subunit	-8.76084	1278.78	0.691827
PGSC0003DMG400042093	Chloroplast photosystem II subunit X	-8.75745	3408.64	2.56972
PGSC0003DMG400000926	Oxygen-evolving enhancer protein 2, chloroplastic	-8.62386	406.064	0.575326
PGSC0003DMG400021144	Photosystem I subunit III	-8.5799	33.5257	1.93521
PGSC0003DMG400007536	Photosystem II reaction center W protein, chloroplastic	-8.45247	3299.31	0.482768
PGSC0003DMG400002626	Photosystem I psaH protein	-7.95615	47.521	1.19574
PGSC0003DMG400022022	Photosystem I reaction center subunit IV B isoform 2	-7.84278	2958.09	0.900403
PGSC0003DMG400008488	Chloroplast pigment-binding protein CP29	-7.78323	4448.68	12.6995
PGSC0003DMG400009268	Proteinase inhibitor	-7.22168	3712.19	6.67259

PGSC0003DMG400020154	Photosystem I reaction center subunit IV A isoform 2	-6.57684	1318.1	0.187907
PGSC0003DMG400022241	Photosystem II 10 kDa polypeptide, chloroplastic	-6.43741	2982.9	0.103778
PGSC0003DMG400044366	Photosystem Q(B) protein	-5.80433	5033.97	11.6048
PGSC0003DMG400018360	Photosystem II 11 kDa protein	-5.6571	817.139	14.6224
PGSC0003DMG400017848	Photosystem Q(B) protein	-5.31259	1.32282	0
PGSC0003DMG400017258	Photosystem II D2 protein	-5.00181	2492.21	0.521304
PGSC0003DMG400001746	Photosystem II D2 protein	-4.80594	1308.32	0.289682
PGSC0003DMG400012033	Photosystem I P700 chlorophyll a apoprotein	-4.46107	1283.31	0.173717
PGSC0003DMG400015960	Photosystem I P700 chlorophyll a apoprotein A1	-4.1147	2131.27	8.58223
PGSC0003DMG400005372	Photosystem I P700 chlorophyll a apoprotein A1	-3.90944	3319.51	7.67041
PGSC0003DMG400017010	Photosystem II reaction center protein J	-2.59883	1670.26	17.4968
PGSC0003DMG400010498	Photosystem II 5 kDa protein, chloroplast	-1.47867	5.29935	1.90151
PGSC0003DMG400015861	Photosystem Q(B) protein	-inf	1.08225	0
PGSC0003DMG400004211	Photosystem Q(B) protein	-inf	1122.64	2.93382
PGSC0003DMG400029115	Photosystem II D2 protein	-inf	1063.64	0.199454
PGSC0003DMG400013010	24K germin	-inf	1.93928	0

Table S5. ChIP-seq and sequential ChIP-seq data generated from potato DM.

SRA accession number	Species	Tissue	Treatment	ChIP type	Antibody	Total # reads processed	% reads mapped	# reads uniquely mapped	% reads uniquely mapped
SRR5242698	Potato DM	Tuber	4ºC for 14 days	Regular ChIP	H3K27me3	11,194,606	85.8%	9,079,846	81.1%
SRR5242697	Potato DM	Tuber	4ºC for 14 days	Regular ChIP	H3K4me3	11,384,997	72.9%	7,708,429	67.7%
SRR7134651	Potato DM	Tuber	4ºC for 14 days	Regular ChIP	H3K4me1	13,357,208	99.5%	11,332,428	84.8%
SRR5242695	Potato DM	Tuber	4ºC for 14 days	Regular ChIP	H4K5,8,12,16ac	6,247,860	67.3%	3,939,314	63.1%
SRR6837805	Potato DM	Tuber	4ºC for 14 days	Regular ChIP	Input	12,158,511	97.2%	9,119,174	75.0%
SRR5242693	Potato DM	Tuber	RT for 14 days	Regular ChIP	H3K27me3	12,106,160	82.5%	9,939,574	82.1%
SRR5242692	Potato DM	Tuber	RT for 14 days	Regular ChIP	H3K4me3	9,785,995	78.8%	6,938,647	70.9%
SRR7134650	Potato DM	Tuber	RT for 14 days	Regular ChIP	H3K4me1	14,946,374	95.7%	11,344,797	75.9%
SRR5242690	Potato DM	Tuber	RT for 14 days	Regular ChIP	H4K5,8,12,16ac	12,336,687	81.2%	9,272,495	75.2%
SRR6837804	Potato DM	Tuber	RT for 14 days	Regular ChIP	Input	29,678,352	98.1%	22,373,033	75.4%
SRR5242688	Potato DM	Leaf	None	Regular ChIP	H3K27me3	10,654,473	89.6%	7,670,728	72.0%
SRR6837803	Potato DM	Leaf	None	Regular ChIP	H3K4me3	10,895,866	73.3%	5,888,397	54.0%
SRR6837801	Potato DM	Leaf	None	Regular ChIP	Input	33,171,563	98.2%	26,022,059	78.4%
SRR6837800	Potato DM	Tuber	RT for 14 days	Sequential ChIP	H3K27me3-H3K4me3	63,675,757	80.3%	1,215,705	2.2%
SRR6837799	Potato DM	Tuber	RT for 14 days	Sequential ChIP	H3K4me3-H3K27me3	13,163,179	78.7%	4,422,831	33.6%
SRR6837798	Potato DM	Tuber	RT for 14 days	Sequential ChIP	H3K27me3-no antibody (control)	6,014,774	21.0%	100,402	1.7%
SRR6837797	Potato DM	Tuber	RT for 14 days	Sequential ChIP	H3K4me3-no antibody (control)	19,378,050	47.7%	2,271,803	11.7%
SRR6837796	Potato DM	Tuber	RT for 14 days	Sequential ChIP	Input	23,825,594	98.4%	17,075,758	71.7%
SRR6837785	Potato DM	Tuber	4ºC for 14 days	Sequential ChIP	H3K27me3-H3K4me3	33,508,754	72.3%	5,483,569	16.4%
SRR6837784	Potato DM	Tuber	4ºC for 14 days	Sequential ChIP	H3K4me3-H3K27me3	17,613,959	86.0%	7,379,338	41.9%
SRR6837787	Potato DM	Tuber	4ºC for 14 days	Sequential ChIP	H3K27me3-no antibody (control)	21,667,896	42.6%	2,866,744	13.2%
SRR6837786	Potato DM	Tuber	4ºC for 14 days	Sequential ChIP	H3K4me3-no antibody (control)	19,144,852	79.1%	7,043,867	36.8%
SRR6837781	Potato DM	Tuber	4ºC for 14 days	Sequential ChIP	Input	16,480,971	97.2%	11,604,748	70.4%

Regular ChIP: ChIP experiments using single antibody

Sequential ChIP: ChIP experiments using 2 antibodies in a sequential order

Table S6. Expression of the genes associated with trimethylation of H3K27 in RT and 14-day cold treated potato tubers.

Gene	Species	Tissue	Locus	Expression in RT (FPKM)	Expression in 4ºC (FPKM)	Up-/Down-regulated	Differentially expressed
Writers							
Putative *CLF*	Potato	Tuber	PGSC0003DMG400034096	4.86	20.01	Up	Yes
Putative *SWN*	Potato	Tuber	PGSC0003DMG400009044	43.43	4.71	Down	Yes
Putative *MEA*	Potato	Tuber	PGSC0003DMG400004008	0.00	0.04	No	No
Erasers							
Putative *REF6*	Potato	Tuber	PGSC0003DMG400015167	7.11	6.75	No	No
Readers							
Putative *LHP1*	Potato	Tuber	PGSC0003DMG400031514	9.92	14.63	Up	Yes
Other PcG components							
Putative *FIE*	Potato	Tuber	PGSC0003DMG401019226	26.78	12.60	Down	Yes
Putative *EMF2*	Potato	Tuber	chr03:37670960..37700107	7.62	9.67	No	No
Putative *MSI1*	Potato	Tuber	PGSC0003DMG400023743	18.54	32.59	Up	Yes
Putative *VRN5*	Potato	Tuber	PGSC0003DMG400027427	5.36	15.45	Up	Yes
Putative *VIN3*	Potato	Tuber	PGSC0003DMG400009357	0.10	0.10	No	No*
Putative *VEL1*	Potato	Tuber	PGSC0003DMG400007693	3.73	18.66	Up	Yes

* Not enough alignments for testing significance.

Table S7. Expression of the genes associated with trimethylation of H3K4 in RT and 14-day cold treated potato tubers.

Gene	Species	Tissue	Locus	Expression in RT (FPKM)	Expression in 4ºC (FPKM)	Up-/Down-regulated	Differentially expressed
Writers							
Putative *ATX1*	Potato	Tuber	PGSC0003DMG400018162	2.99	3.32	No	No
Putative *ATXR3*	Potato	Tuber	PGSC0003DMG400026163	12.44	16.50	Up	Yes
Putative *ATXR7*	Potato	Tuber	PGSC0003DMG400026863	15.73	7.20	Down	Yes
Erasers							
Putative *JMJ14*	Potato	Tuber	PGSC0003DMG400011477	18.20	16.38	No	No
Putative *JMJ15*	Potato	Tuber	PGSC0003DMG400021194	1.03	0.71	No	No
Readers							
Putative *VAL1*	Potato	Tuber	PGSC0003DMG400030567	0.80	0.34	No	No
Putative *ORC1A*	Potato	Tuber	PGSC0003DMG401012812	11.21	36.53	Up	Yes
Putative *ORC1B*	Potato	Tuber	PGSC0003DMG402012812	4.80	23.22	Up	Yes
Putative *ING1*	Potato	Tuber	PGSC0003DMG400011588	25.00	14.44	Down	Yes
Putative *ING2*	Potato	Tuber	PGSC0003DMG400008367	14.35	26.18	Up	Yes
Putative *AL1*	Potato	Tuber	PGSC0003DMG400023078	31.37	50.32	Up	Yes
Putative *WDRa5*	Potato	Tuber	PGSC0003DMG401030621	15.09	9.30	Down	Yes

Table S8. Primers used for qRT-PCR.

Genes	Species	Forward	Reverse
Putative *CLF*	Potato	CGGAAGGGTGACAAACTAAAA	TGGCAAAGATACCAACTCTGT
Putative *MEA*	Potato	AGAAAAGGACAGAAGAGAATGC	GGAACCAATCCCAGTTTGC
Putative *SWN*	Potato	AGCAGATAAGCGGGGGAAA	TGGGTTTGATGAGTGATTGG
Putative *REF6*	Potato	GAGGAGTGTCAAGGTCAAAAGG	TGCCAAGCAAACCAACTAAA
Putative *LHP1*	Potato	AGCTCAAGGCTTCTGTTGGA	CCGGCATCTTCAGGATCTT
Putative *FIE*	Potato	CAACCCTTGAAACCATCTCTT	CTCCAGCACCAGCAAATACC
Putative *EMF2*	Potato	GAATTACACTTGCTACTGCA	CCAACGCCGTGCTGTTAGA
Putative *MSI1*	Potato	TGGGGACTCATACATCGGAAA	TCATCATAGTGTCGGGCATC
Putative *VRN5*	Potato	GCAAGGCTGTTTAGAGGGATT	GGCGAGAAGTAGATGGTTTCC
Putative *VIN3*	Potato	GCACTTGAGCACGAACAATC	CAACTCTTCTCGCCTCCTTT
Putative *VEL1*	Potato	GCGGAAAGGAAAATCAGAAC	TGTCCACCACACTCTAACCA
Putative *ATX1*	Potato	ATGGCTGTGAAACGGAAAAC	CTTGAACTTGGGGCAGAAAA
Putative *ATXR3*	Potato	ATAAACAACCCTGGCGAAGA	ACAAACTGAAAAACGCTCCA
Putative *ATXR7*	Potato	TTCTTTGTGCTTGTCGCTTG	TTTGGGTTCAGTTCGGAGAG
Putative *JMJ14*	Potato	CGAAAGATACTGCGGGAAAG	GACGAACCTCCAACGAAAGA
Putative *JMJ15*	Potato	TGTTCTCGTCCGTTACAGCA	CATTTCCAATCCATCCCTCT
Putative *VAL1*	Potato	TCAAGAGAGCAGGATGGTTG	AGGCATTGGAAGAGACGAAA
Putative *ORC1A*	Potato	TGCATTGACCTCTGTGCTTC	CGGCGTACTTTCACCAAAAT
Putative *ORC1B*	Potato	TGTGAACCTGGAGTGAAGCA	ATACCGAGCCAACCACGAC
Putative *ING1*	Potato	CAAAAGAAGCGGAAAGGAAA	CAAGAAAACAGCCAACACAGA
Putative *ING2*	Potato	CTGCTATTCCTTCCGCATCA	CCGACTACCCTTTTTCACCA
Putative *AL1*	Potato	GAGCCAGCACTTGGGATAAA	GAAAGCCACAGAAAGCAACC
Putative *WDRa5*	Potato	CGGAAGGAAACAAGAAACCA	ACTGGAGGAAGAATGCCAAA
EF1α	Potato	ATTGGAAACGGATATGCTCCA	TCCTTACCTGAACGCCTGTCA
