# Supplementary material for: Cold stress induces enhanced chromatin accessibility and bivalent histone modifications H3K4me3 and H3K27me3 of active genes in potato
Source: Genome Biol. 2019 Jun 17;20:123. doi: 10.1186/s13059-019-1731-2 (PMC6580510; doi:10.1186/s13059-019-1731-2)
Supplement: Supplementary file 3 — Review history. (DOCX 1242 kb) [file 13059_2019_1731_MOESM3_ESM.docx]

Review History

**First round of review**

**Reviewer 1**

**Are you able to assess all statistics in the manuscript, including the appropriateness of statistical tests used?**

Yes, and I have addressed all statistics in my report.

**Comments to author:**

This manuscript describes a detailed analysis of the genome wide detection and mapping of histone modifications leading to chromatin remodelling under the influence of abiotic stress conditions in potato. Potato cold storage issues relating to cold sweetening, while biochemically quite well understood still represents a significant problem. This work has take a novel and important approach to illustrate the higher levels of gene regulation that appear to influence this trait. The manuscript is well written and describe all the experiments clearly. The conclusions are measured and appropriate. The results described will be of broad interest to wide readership interested in plant genomics.

While many potential experiments could be suggested to verify and validate the results obtained and described here, the dataset and conclusions to be drawn represents a valuable scientific contribution to the field.

**Reviewer 2**

**Are you able to assess all statistics in the manuscript, including the appropriateness of statistical tests used?**

No, I do not feel adequately qualified to assess the statistics.

**Comments to author:**

In this manuscript, authors examined genome-wide changes in chromatin landscape in Potato tuber in cold storage. Genomic analysis was based on DNase I hypersensitive site (DHS)-Seq, H3K27me3 ChIP-Seq and H3K4me3 ChIP-Seq. Main conclusion was that "bivalent" regions of H3K4me3/H3K27me3 are associated with differentially expressed (both up-regulated and down-regulated) loci upon cold storage. Overall, analysis was very thorough and found an interesting chromatin landscape changes upon cold treatment. Overreaching conclusion was that the cold-responsive genes are bivalent and more readily accessible for gene regulation, perhaps enabling plants to respond to stress more efficiently.

1) why 14 days of cold? Are these differentially expressed and/or bivalent/higher accessible loci respond to cold quickly? Does this change recover upon the return to warm temperature?

2) Is there any difference between cold-treated tubers and leaves (beside obvious difference in photosynthesis/carbohydrate metabolisms)? Authors proposed a model as a general mechanism in cold-responses, and therefore authors should examine whether similar chromatin landscape occur in above ground tissues, in my opinion.

**Authors Response**

**Point-by-point responses to the reviewers’ comments:**

Reviewer #1: This manuscript describes a detailed analysis of the genome wide detection and mapping of histone modifications leading to chromatin remodelling under the influence of abiotic stress conditions in potato. Potato cold storage issues relating to cold sweetening, while biochemically quite well understood still represents a significant problem. This work has take a novel and important approach to illustrate the higher levels of gene regulation that appear to influence this trait. The manuscript is well written and describe all the experiments clearly. The conclusions are measured and appropriate. The results described will be of broad interest to wide readership interested in plant genomics.

While many potential experiments could be suggested to verify and validate the results obtained and described here, the dataset and conclusions to be drawn represents a valuable scientific contribution to the field.

*Response: We appreciate the comments from the reviewer. We are currently conducting additional experiments in potato as well as in model plant Arabidopsis thaliana to generate more information for further understanding of chromatin dynamics in response to cold stress, especially the presence of bivalent H3K4me3-H3K27me3 mark in different plant species and under different stress conditions. In addition, we have initiated experiments using relevant Arabidopsis mutants (for example clf28, atx1-2 and lhp1) to explore molecular mechanism associated with what we discovered in potato. We will soon develop our second manuscript in this exciting field of chromatin dynamics associated abiotic stresses in plants.*

Reviewer #2: In this manuscript, authors examined genome-wide changes in chromatin landscape in Potato tuber in cold storage. Genomic analysis was based on DNase I hypersensitive site (DHS)-Seq, H3K27me3 ChIP-Seq and H3K4me3 ChIP-Seq. Main conclusion was that "bivalent" regions of H3K4me3/H3K27me3 are associated with differentially expressed (both up-regulated and down-regulated) loci upon cold storage. Overall, analysis was very thorough and found an interesting chromatin landscape changes upon cold treatment. Overreaching conclusion was that the cold-responsive genes are bivalent and more readily accessible for gene regulation, perhaps enabling plants to respond to stress more efficiently.

*Response: We very much appreciate the positive comments from the Reviewer. In the revised manuscript, we have toned down our conclusion on the relationships among the bivalent H3K4me3-H3K27me3 mark, chromatin accessibility and gene expression throughout the revised manuscript.*

1) why 14 days of cold?

*Response: Cold-induced sweetening (CIS) in potato tubers is strongly correlated with the activity of a key enzyme vacuolar acid invertase (VINV), which catalyzes sucrose into reducing sugars fructose and glucose. The expression of VInv gene is significantly up-regulated in tubers stored under cold temperature (4°C) and reaches to the peak after approximately 14 days (Bhaskar et al., 2010, Plant Physiology, 154: 939-948.). Thus, we chose to treat potato tubers under 4°C for 14 days to simulate an ideal condition for potato CIS. We have added this information in the Methods section of the revised manuscript.*

Are these differentially expressed and/or bivalent/higher accessible loci respond to cold quickly? Does this change recover upon the return to warm temperature?

*Response: These are excellent questions. Our data for chromatin accessibility, histone modifications and gene expression were obtained from potato tubers, which were separated and treated at 22°C and 4°C for 14 days, respectively. Thus, unfortunately our current data sets cannot address how fast the differentially expressed and/or bivalent/higher accessible loci respond to cold. This question will need to be addressed by experiments based on a time-course manner. We appreciate the question on the recovering of cold-induced transcriptional and epigenetic changes under warm temperature. The minimum expression of VInv under warm temperature is well known. In fact, the processing industry typically moves cold-stored tubers to a warm room, which is called “re-conditioning”, to “burn” the reducing sugars and to suppress the VInv. Nevertheless, we agree with the Reviewer that a scientific answer to the question related to “re-conditioning” will need to be from experiments based on also a time-course manner. We are planning to do these experiments on a genome-wide scale, which will require a significant budget as well as time, and will be described in a separate manuscript.*

2) Is there any difference between cold-treated tubers and leaves (beside obvious difference in photosynthesis/carbohydrate metabolisms)?

*Response: We assume that the Reviewer’s question is about the difference “between cold-treated tubers and leaves”, not “between cold-treated tubers and cold-treated leaves” (since we don’t have data from cold-treated leaves). To address this question, we compared differentially expressed genes (DEGs) that associated with specific DHSs between cold-treated tubers and leaves on a genome-wide scale. We found that up-regulated genes in cold-treated tubers were more frequently associated with cold tuber-specific DHSs located in all tested genomic regions compared to the association of up-regulated genes in leaves with leaf-specific DHSs (z-test p<0.0002) (Fig. 1). This association pattern is different from the one between RT and cold tubers, which showed a similar frequency of association with temperature-specific DHSs location in putative promoters (1kb upstream of the TSS) and intergenic regions.*

*Figure 1. Comparison between up-regulated genes in cold tubers associated with cold tubers-specific DHSs and up-regulated genes in leaves associated with leaf-specific DHSs at different genomic locations, including exons, introns, TSS1k (1 kb upstream regions of TSSs), TSS200 (200 bp upstream regions of TSSs), TTS1k (1 kb downstream regions of TTSs), TTS200 (200 bp downstream regions of TTSs) and intergenic regions (1-5 kb upstream regions of TSSs or 1-5 kb downstream regions TTSs). *z-test p<0.0002.*

*For histone modification H3K27me3, we observed that active genes (FPKM>1 in both cold-treated tubers and leaves; n=15,103) displayed significant enrichment of H3K27me3 in cold-treated tubers (Wilcoxon Rank Sum test, p<2.2e-16) compared to those in leaves (Fig. 2a). In contrast, constitutively silenced genes (FPKM=0 in either cold-treated tubers or leaves; n=12,124) as well as random intergenic regions (n=12,124) showed lower H3K27me3 levels in cold-treated tuber than in leaves (Fig. 2a). The overall pattern of H3K27me3 levels between cold-treated tubers and leaves is similar to that between RT and cold tubers. Differing from the H3K4me3 pattern found between RT and cold tubers, active genes displayed significant lower levels of H3K4me3 in cold-treated tubers than in leaves, particularly at 5’ end regions (Wilcoxon Rank Sum test, p<2.2e-16) (Fig. 2b), while both constitutively silenced genes and random intergenic regions showed slightly higher levels of H3K4me3 in cold-treated tubers than in leaves, including their flanking regions at both sides (Fig. 2b).*

*Figure 2. Comparison of histone modification levels between cold-treated tubers and leaves. (a) The ratio of H3K27me3 signal intensity between cold-treated tubers and leaves. (b) The ratio of H3K4me3 signal intensity between cold-treated tubers and leaves. The same set of active genes (n=15,103) and constitutively silenced genes (n=12,124) were used in all analyses. Each active gene showed transcription (FPKM >1) in both cold-treated tubers and leaves. Constitutively silenced genes did not show transcription in either cold-treated tubers and leaves (FPKM=0). “Random intergenic regions” were randomly selected from regions that were at least 2 kb away from any annotated genes. The length and number of the random intergenic regions were the same as constitutively silenced genes. Genes were divided into 100 bins and aligned together from TSSs to TTSs. Genes flanking regions (±1 kb) were analyzed in 100 bins. Histone modification signal was measured by quantifying the mid-point of pair-end ChIP-seq reads within each bin and normalizing by ChIP-seq read number per bp genome region per million mapped reads.*

*For DNase I sensitivity, active genes showed higher DNase I sensitivity across the gene body regions in cold-treated tubers compared to those in leaves (Wilcoxon Rank Sum test, p<2.2e-16) (Fig. 3). However, their flanking regions at both ends displayed lower DNase I sensitivity in cold-treated tubers than in leaves (Fig. 3). In addition, similar higher levels in chromatin accessibility were also observed across the gene body regions of constitutively silenced genes (Wilcoxon Rank Sum test, p<2.2e-16). The pattern of the DNase I sensitivity between cold-treated tubers and leaves is similar to that between RT and cold tubers.*

*We decided to not include these analyses in the revised manuscript since we wish to focus our manuscript on the chromatin dynamics between RT and cold tubers.*

*Figure 3. Comparison of DNase I sensitivity for genes with different expression levels (FPKM) between cold-treated tubers and leaves. Each active gene showed transcription (FPKM >1) in both cold-treated tubers and leaves. Constitutively silenced genes did not show transcription in either cold-treated tubers and leaves (FPKM=0). Genes were divided into 100 bins and aligned together from TSSs to TTSs. Gene flanking regions (±1 kb) were also analyzed in 100 bins.*

Authors proposed a model as a general mechanism in cold-responses, and therefore authors should examine whether similar chromatin landscape occur in above ground tissues, in my opinion.

*Response: We would like to thank the Reviewer’s comment. Following the Reviewer’s suggestion, we have adjusted our model specifically for potato tubers, since the cold-induced bivalent H3K4me3-H3K27me3 mark was discovered in tubers. In addition, we have also included potato leaves (RT condition). In both RT tubers and leaves, active genes display similar association patterns of H3K4me3 (enriched at the 5’ ends of active genes), while inactive genes are associated with H3K27me3 (enriched in gene bodies of repressed genes). In contrast, only the active genes in cold tubers are associated with the bivalent H3K4me3-H3K27me3 mark as well as greater chromatin accessibility. These features are now included in the revised model.*

**Second round of review**

**Reviewer 2**

I am satisfied with this revision. No further comment.
